# Supplementary material for: Rural-urban disparities in the nutritional status of younger adolescents in Tanzania
Source: PLoS One. 2021 Dec 20;16(12):e0261480. doi: 10.1371/journal.pone.0261480 (PMC8687541; doi:10.1371/journal.pone.0261480)
Supplement: S1 File — (DOC) [file pone.0261480.s001.doc]

Moshi Caregiver Health Survey

Harvard Medical School, Kilimanjaro Christian Medical Centre (KCMC) and National Bureau of Statistics (Tanzania)

August 2004

|  |  |  |  |
| --- | --- | --- | --- |
| Consent ID |  |  |  |
| Subject ID |  | Date of Assessment  - -/- -/- - - - |  |
| Interviewer ID |  | Date Edited by Int  - -/- -/- - - - |  |
| Supervisor ID  - - - |  | Date Edited by Sup  - -/- -/- - - - |  |
| Editor ID  - - - |  | Date Checked by Edi  - -/- -/- - - - |  |
| Data entry ID  - - - |  | Date entered  - -/- -/- - - - |  |
| Time began  - -.- - |  | Time ended  - -.- - |  |

| **Section** | **Question number** | **Page number** |
| --- | --- | --- |
| 1. Demographics + Income Sources | 1-16 | 3-4 |
| 2. Socio-economic status - HH | 17-18 | 5-7 |
| 3. Your health | 19-39 | 7-13 |
| 4. Your child’s health | 40-50 | 14-15 |
| 5. Strengths and disabilities | 61 | 17-18 |
| 6. Challenges of raising a teenager | 62-86 | 19-23 |
| 7. Parental efficacy | 87-96 | 24-25 |
| 8. Child Activities |  |  |
| 9. Child Self-Efficacy |  |  |
|  |  |  |

| 1a | Have there been any changes in your family since the last CHASE survey?  **Comments:** | | | QN1ASES |
| --- | --- | --- | --- | --- |
| 1b | In the past 12 months, how difficult has it been to meet your family’s basic needs? | Very difficult  A little difficult  Not at all difficult | 01  02  03 | QN1BSES |
|  | Maoni: | | | QN1BSESP |

**Demographics and Income Sources:**

|  |  |  |  |  | Health Status | ***For Children less than 18 years*** | | | | | | |
| --- | --- | --- | --- | --- | --- | --- | --- | --- | --- | --- | --- | --- |
| Member ID | Can you please list the first names of all household members?**    Name | Sex    M…1  F….2 | How old is [name]?   Age | Relation to Household Head | How is [name’s] health?  Excellent..…1  Good……….2  Not Good…..3  Poor………..4 | Is [name’s] mother alive?  If ‘No’ go to 16b  If ‘DK’ go to 17  Yes…1  No….2  DK…8 | If mother is alive:  Does [name’s} mother live in this household?  Yes…1  No….2 | If mother is not alive:  What year did [name’s] mother die?* | Is [name’s] father alive?  If ‘No’ go to 16b  If ‘DK’ go to 17  Yes…1  No….2  DK…8 | If father is alive:  Does [name’s} father live  in this household?  Yes…1  No….2 | If father is not alive:  What year did [name’s] father die?* |  |
|  | 2 | 3 | 4 | 5 | 13 | 16 | 16a | 16b | 17 | 17a | 17b |  |
| 01 |  |  |  |  |  |  |  |  |  |  |  |  |
| 02 |  |  |  |  |  |  |  |  |  |  |  |  |
| 03 |  |  |  |  |  |  |  |  |  |  |  |  |
| 04 |  |  |  |  |  |  |  |  |  |  |  |  |
| 05 |  |  |  |  |  |  |  |  |  |  |  |  |
| 06 |  |  |  |  |  |  |  |  |  |  |  |  |
| 07 |  |  |  |  |  |  |  |  |  |  |  |  |
| 08 |  |  |  |  |  |  |  |  |  |  |  |  |
| 09 |  |  |  |  |  |  |  |  |  |  |  |  |
| 10 |  |  |  |  |  |  |  |  |  |  |  |  |

** Household member is a person who has stayed in the house for at least six months and who shares food from the same bowl as other household members*.

| **Relation to Household Head code:**  Household head…….1 1  Spouse 2  Son/daughter 3  Father/mother 4  Sister/brother 5  Son/daughter-in-law 5  Grand child 7  Brother/sister-in-law 8  Other relative 9  Non-relative 10 | **Marital Status code:**  Never married………..1  Married ….2  Separated ….3  Divorced ….4  Widow/widower ….5 | **Religion code:**  Christian………………1  Muslim………….…….2  Hindu…………………3  Other non-Christian..…4  None………………….5    **Ethnic Group code:**  Chagga……..………...1  Pare ….2  Asian ….3  Arab ….4  Other………………...5 | **Education code:**  Chekechea……………00  Primary 1……………..01  Primary 2……………..02  Primary 3……………..03  Primary 4……………..04  Primary 5……………..05  Primary 6……………..06  Primary 7……………..07  Secondary 1…………..08  Secondary 2…………..09  Secondary 3…………..10  Secondary 4…………..11  Secondary 5…...……...12  Secondary 6…………..13  Certificate.……………14  Diploma………..…….15  University……………16 | **Primary Source of Income code:**  Farming, forestry, fishing………….……....1  Non-agri day labor………….……………...2  Professional/Clerical…………………….....3  Service (tailor, hairdresser, mechanic)….….4  Domestic servant.………………………..…5  Vendor (vegetable, products)……………....6  Beggar ……………………….….7  Mining…..………………………..………...8  Commercial sex worker……………..….…..9  Work in a bar………………………………10  Not employed………………………………11  Business ……………………………………12  Other………………………………….……..13 |
| --- | --- | --- | --- | --- |

*If doesn’t know date of death write 2098

SOCIO-ECONOMIC STATUS OF HOUSEHOLD

|  |  |  |  |  |
| --- | --- | --- | --- | --- |
| 19a | Have you moved in the last year? If yes, when? | Yes  Date: (mm/yyyy)  No  DK  Refused | 01  __  02  03  04 | QN19ASES QN19ASED  QN19ASES |
| 19b | How long have you lived here? | **(Write no. of years)**  Don’t know  Refused | __  98  99 | Q18ASES1 Q18ASES1 |
|  |  |  |  |  |
|  |  |  |  |  |
| 19c | With whom does your child spend MOST of his/her time? | By his/herself  Two parents  One parent—mother only  One parent—father only  House servant (ayah)  Other adult relative (grandparents, aunt, uncle)  Other adult not related to child (foster parent, step-parent, friend, etc)  Other youth (brothers, sisters, step-siblings, friends, etc) | 01  02  03  04  05  06  07  08 | QN19CSES |
| 19d | Do you or someone in your family own or rent the house you are living in? | Respondent owned  Rented  Someone in family owns  Someone in the family rents  Don’t know  Refused | 01  02  03  04  98  99 | Q18BSES2 Q18BSES2 |
| 19e | What is the material of your floor in the main part of your household? | Earth/ sand  Cement  Other **(specify)**  _________________ | 01  02  03 | Q18CSES3 Q18CSES3  Q18CSES3S Q18CSES3SP |
| 19f | What is the main source of drinking water for your household? | Piped into dwelling  Public tap  Neighbour’s house  Pond, river stream  Other **(specify)**  _________________  Don’t know | 01  02  03  04  05  98 | Q18DSES4 Q18DSES4  Q18DSE4S Q18DSES4SP |
| 19g | What type of toilet facility does your household use? | Flush to sewage/septic tanks  Pour flush latrine  Improved pit latrine  Traditional pit latrine  Other **(specify)** _________________  Don’t know | 01  02  03  04  05  98 | Q18ESES5 Q18ESES5  Q18ESE5S Q18ESES5SP |
| 19h | Is this facility located within your dwelling, or yard or compound? | Yes, within dwelling  Yes, within yard/ compound  No, outside yard/compound  Other **(specify)** _________________ | 01  02  03  04 | Q18FSES6 Q18FSES6  Q18FSE6S Q18FSES6SP |
| 19i | Does your household have?  **(Mark all that apply: 1= yes, 2=no)** | Electricity | 1 2 | Q18GSES6 |
|  |  | A radio | 1 2 | Q18GSES1 |
|  |  | A landline phone | 1 2 | Q18GSES2 |
|  |  | A television | 1 2 | Q18GSES3 |
|  |  | A refrigerator | 1 2 | Q18GSES4 |
| 19j | Does anyone in your household have a mobile phone? | Yes  No | 1 01  2 02 | Q18HSES7 |
| 19k | Does any member of your household own:  **(Mark all that apply: 1=yes, 2=no)** | A bicycle  A motorcycle  A car or truck | 1 2  1 2  1 2 | Q18ISES**8**  Q18ISES**1**  Q18ISES**2** |
| 19l  19m | Do you have any children (< 18 yrs) living outside this household?  For how many children does the household pay school fees / school  contributions?  **(Include all children- both resident and non-resident)** | Yes  No | 1  2 | Q18KSE10 Q18KSES10 |
|  |  | **(If yes, how many children?)** | ____ | Q18KS10S Q18KSES10SP |
|  |  | Number of children | ____ | Q18LSE11 Q18LSES11 |
|  |  |  |  |  |
|  |  |  |  |  |
|  |  |  |  |  |
|  |  |  |  |  |

**Your health**

I am now going to ask you a few questions about your own health.

| **20** | Just thinking about your **physical health** during the **PAST MONTH**, including being sick or injured, on how many days during the past month was your physical health NOT good? | | (days)  (0-30) | **___** | Q19YH1 Q19YH1 |
| --- | --- | --- | --- | --- | --- |
| **21** | And how about your **mental health**, which includes stress, depression, and problems with emotions, on how many days during the past month was your mental health NOT good? | | (days)  (0-30) | **___** | Q20YH2 Q20YH2 |
| **22** | During the past month, on how many days did poor physical or mental health keep you from doing your usual activities? | | (days)  (0-30) | **___** | Q21YH3 Q21YH3 |
| **23** | Do you think your weight is? | About right  I need to gain weight  I need to loose weight  Not sure | | 1  2  3  4 | Q22YH4 Q22YH4 |
| **24** | Have you **ever** been diagnosed with any serious disease such as asthma, diabetes, or a heart condition? | Yes……Continue to Q25  No…… Go to Q26 | | 1  2 | Q23YH5 Q23YH5 |

| **25** | **What were you diagnosed with? (List each separately)**  **a.________________________________________________________________________________________________** | Q24YH6A Q24YH6A |
| --- | --- | --- |
|  | **b.________________________________________________________________________________________________** | Q24YH6B Q24YH6B |
|  | **c.________________________________________________________________________________________________** | Q24YH6C Q24YH6C |
|  | **d.________________________________________________________________________________________________** | Q24YH6D Q24YH6D |

Now I would like you to just think about the past 12 months, that is since (refer to month one year ago). Please tell me how often you have had any of the following problems:

| 26 | In the past year, how often have you had a cold or flu? Would you say…. | Never……  Once or twice…….  Three or more times…. | 1  2  3 | Q25YH7 Q25YH7 |
| --- | --- | --- | --- | --- |
| 27 | How often have you had malaria? Would you say………….. | Never……  Once or twice…….  Three or more times…. | 1  2  3 | Q26YH8 Q26YH8 |
| 28 | How often have you had a headache? Would you say…. | Never……  Once or twice…….  Three or more times…. | 1  2  3 | Q27YH9 Q27YH9 |
| 29 | How often have you had an upset stomach with vomiting, diarrhea or a fever? Would you say… | Never……  Once or twice…….  Three or more times…. | 1  2  3 | Q28YH10 Q28YH10 |
| 30 | Overall, would you say in general your health is……….. | Poor…………………….  Fair……………………..  Good……………………  Excellent………………. | 1  2  3  4 | Q29YH11 Q29YH11 |
| 31 | (**If male)** Is your wife pregnant?  **(If female)** Are you currently pregnant? | Yes  No  I don’t know  Refused | 1  2  98  99 | Q30YH12 Q30YH12 |

| **32. HOPKINS SYMPTOM CHECKLIST**  Listed below are some symptoms or problems that people sometimes have. Please listen to each one carefully and decide how much the symptoms bothered or distressed you in the last week, including today.) | | | | | | | |
| --- | --- | --- | --- | --- | --- | --- | --- |
| **Anxiety Symptoms** | **Not at all**  **(1)** | **A Little**  **(2)** | **Quite a bit**  **(3)** | **Extremely**  **(4)** | **Don’t**  **Know**  **(8)** | **Code name** | |
| a) Suddenly scared for no reason |  |  |  |  |  | Q31SANX1 | Q31ASANX1 |
| b) Feeling fearful  (to be in fear) |  |  |  |  |  | Q31SANX2 | Q31BSANX2 |
| c) To feel faintness, dizzy, or weakness |  |  |  |  |  | Q31SANX3 | Q31CSANX3 |
| d) To feel nervous |  |  |  |  |  | Q31SANX4 | Q31DSANX4 |
| e) Heart beating rapidly or pounding more than usual |  |  |  |  |  | Q31SANX5 | Q31ESANX5 |
| f) To tremble/trembling |  |  |  |  |  | Q31SANX6 | Q31FSANX6 |
| g) To feel tense, nervous, or not calm |  |  |  |  |  | Q31SANX7 | Q31GSANX7 |
| h) Headache |  |  |  |  |  | Q31SANX8 | Q31HSANX8 |
| i) To have periods of fear or panic |  |  |  |  |  | Q31SANX9 | Q31ISANX9 |
| j) To feel anxious, such that you are restless, can’t sit still |  |  |  |  |  | Q31SAN10 | Q31JSANX10 |

| **Depression Symptoms** | | **Not at all**  **(1)** | **A Little**  **(2)** | | **Quite a bit**  **(3)** | | **Extremely**  **(4)** | | **DK**  **(8)** | |  |
| --- | --- | --- | --- | --- | --- | --- | --- | --- | --- | --- | --- |
| k) To feel weak and when you find yourself conducting activities slowly |  | |  |  | |  | |  | | Q31SDEP1 Q31KSDEP11 | |
| l) Blaming yourself for things | |  |  | |  | |  | |  | | Q31SDEP2 Q31LSDEP12 |
|  | |  |  | |  | |  | |  | |
| m) To cry easily | |  |  | |  | |  | |  | | Q31SDEP3  Q31MSDEP13 |
|  | |  |  | |  | |  | |  | |
| n) Loss of sexual interest or pleasure | |  |  | |  | |  | |  | | Q31SDEP4 Q31NSDEP14 |
|  | |  |  | |  | |  | |  | |
| o) To not feel like eating (loss of appetite) | |  |  | |  | |  | |  | | Q31SDEP5 Q31OSDEP15 |
|  | |  |  | |  | |  | |  | |
| p) To have hard time falling asleep when it is time to go to bed or staying asleep until morning | |  |  | |  | |  | |  | | Q31SDEP6 Q31PSDEP16 |
| q) Feeling hopeless about the future | |  |  | |  | |  | |  | | Q31SDEP7 Q31QSDEP17 |
|  | |  |  | |  | |  | |  | |
| r) To feel unhappy | |  |  | |  | |  | |  | | Q31SDEP8 Q31RSDEP18 |
|  | |  |  | |  | |  | |  | |
| s) To feel lonely | |  |  | |  | |  | |  | | Q31SDEP9 Q31SSDEP19 |
|  | |  |  | |  | |  | |  | |
| t) Thoughts of taking your own life | |  |  | |  | |  | |  | | Q31SDP10 Q31TSDEP20 |
|  | |  |  | |  | |  | |  | |
| u) Feeling of being trapped or caught | |  |  | |  | |  | |  | | Q31SDP11 Q31USDEP21 |
|  | |  |  | |  | |  | |  | |
| v) Worrying about many things | |  |  | |  | |  | |  | | Q31SDP12 Q31VSDEP22 |
|  | |  |  | |  | |  | |  | |
| w) Feeling no interest in things | |  |  | |  | |  | |  | | Q31SDP13 Q31WSDEP23 |
|  | |  |  | |  | |  | |  | |
| x) Feeling everything is an effort | |  |  | |  | |  | |  | | Q31SDP14 Q31XSDEP24 |
|  | |  |  | |  | |  | |  | |
| y) Feelings of worthlessness | |  |  | |  | |  | |  | | Q31SDP15 Q31YSDEP25 |

**Now I am going to ask you a few questions about Malaria**

| 33a | How often is malaria a problem in your household? | More than 5 times a year  3-5 times a year  1-2 times a year  About once a year or less | 01  02  03  04 | Q32HV1A Q32HV1A |
| --- | --- | --- | --- | --- |
|  |  |  |  |  |
|  |  |  |  |  |
| 33b | Which malarial preventative measures are usually practiced at your home? | None  Use of mosquito/impregnated bednets  Mosquito repellents (including sprays, barks, and creams)  Household spraying (from private company or municipal council)  Malaria prophylaxis  Intermittent presumptive treatment (IPT)  Removal of mosquito breeding sites  Other___________________ | 01  02  03  04  05  06  07  08 | QN33BHV  QN33BHVO |

**Now I am going to ask you a few questions about HIV/AIDS**.

|  |  |  |  |  |
| --- | --- | --- | --- | --- |
| 34 | How can a person find out if she or he has HIV (the illness that causes AIDS)?  (**Circle all mentioned – do not read out)** | Go for a test | 1 2 | Q33HV2A Q33HV2A |
|  |  | Go to a health facility | 1 2 | Q33HV2B Q33HV2B |
|  |  | Go to counseling/testing facility | 1 2 | Q33HV2C Q33HV2C |
|  |  | Go to traditional doctor | 1 2 | Q33HV2D Q33HV2D |
|  |  | Other  **(specify)**  _____________________ | 1 2 | Q33HV2E Q33HV2E  Q33HV2EO |
|  |  |  |  |  |
| 35  36 | Which of the following events would be a good reason for someone to get an HIV test?  **(Ask for each event and mark all that apply)** | Planning to get married | 1 2 | Q35HV4A Q35HV4A |
|  |  | Wanting to have children | 1 2 | Q35HV4B Q35HV4B |
|  |  | Becoming pregnant | 1 2 | Q35HV4C Q35HV4C |
|  |  | Being ill | 1 2 | Q35HV4D Q35HV4D |
|  |  | Planning for the future | 1 2 | Q35HV4E Q35HV4E |
|  |  | Having unprotected sex | 1 2 | Q35HV4F Q35HV4F |
|  |  | Starting a new job | 1 2 | Q35HV4G Q35HV4G |
|  |  | To protect one’s partner | 1 2 | Q35HV4H |
|  |  | To protect one’s children | 1 2 | Q35HV4I |
|  |  | Other  **(specify)**  **________________________________________________** |  | Q35HV4J Q35HV4H  Q35HV4HO --------- |
|  | Which of the following could stop someone from getting a HIV test?  **(Ask for each fear and mark all that apply)** | Fear of losing job | 1 2 | Q36HV5A Q36HV5A |
|  |  | Fear of losing medical benefits | 1 2 | Q36HV5B Q36HV5B |
|  |  | Fear of losing pension | 1 2 | Q36HV5C Q36HV5C |
|  |  | Fear of losing partner | 1 2 | Q36HV5D Q36HV5D |
|  |  | Fear of knowing | 1 2 | Q36HV5E Q36HV5E |
|  |  | Fear of stigma | 1 2 | Q36HV5F Q36HV5F |
|  |  | Other  (specify)_________________________________ |  | Q36HV5G Q36HV5G  Q36HV5GO |
|  |  | Don’t know | 98 |  |
| 37 | Is there anyone in this household who has gone for a HIV/AIDS test? | Yes  No  Don’t know  Refused | 01  02  98  99 | Q37HV6 Q37HV6 |
| 38 | Do you know where to go in Moshi for an HIV/AIDS test? | Yes  No | 01  02 | QN38HV |
| 39 | How strongly do you believe that all adults in the community should be tested for HIV/AIDS? | Strongly agree  Agree  Neither agree nor disagree  Disagree  Strongly disagree | 01  02  03  04  05 | QN39HV |
| 40 | Before moving on to ask about your child’s health, I would like to know if anyone in the household has a drinking problem?  **(Mark all that apply)** | Yes, self | 01 | Q38HV7 Q38HV7 |
|  |  | Yes, spouse | 02 | Q38HV7B |
|  |  | Yes, a grown up child | 03 | Q38HV7C |
|  |  | Yes, other adult relative | 04 | Q38HV7D |
|  |  | Yes, non relative | 05 | Q38HV7E |
|  |  | No **(If no go to Q41)** | 06 | Q38HV7F |
| 41 | How serious a problem is this for that person? | Not serious  A bit serious (interferes with responsibility at home or at work occasionally)  Serious (interferes with responsibilities at home or at work most of the time) | 01  02  03 | Q39HV8 Q39HV8 |

**Your Child’s Health**

 I will begin by asking you some questions about your child’s health.

| 42 | Just thinking about your child’s **physical health** during the **PAST MONTH**, including being sick or injured, on how many days during the past month was your child’s physical health NOT good? | | (days)  (0-30) | **____** | | Q40YCH1 Q40YCH1 | |
| --- | --- | --- | --- | --- | --- | --- | --- |
| 43 | And how about your child’s **mental health**, which includes stress, depression, and problems with emotions, on how many days during the past month was your child’s mental health NOT good? | | (days)  (0-30) | **____** | | Q41YCH2 Q41YCH2 | |
| 44  45 | Katika kipindi cha siku 30 zilizopita, ni siku ngapi udhaifu wa mwili au akili ulimfanya motto wako ashindwe kufanya kazi zake za kawaida?  Do you think your child’s weight is? | (days)  (0-30)  About right  Needs to gain weight  Needs to lose weight  Not sure | | _____  1  2  3  4 | | Q42YCH3 Q42YCH3  Q43YCH4 Q43YCH4 | |
|  |  |  | |  | |  | |
|  |  |  | |  | |  | |
|  |  |  | |  | |  | |
| 56 | Has your child **ever** been diagnosed with any serious disease such as asthma, diabetes, or a heart condition? | Yes……**Continue to Q 57**  No…… **Skip Q 57** | | 1  2 | | Q44YCH5 Q44YCH5 | |
| 57 | **What was your child diagnosed with? (List each separately)**  **a.________________________________________________________________________________________________** | | | | Q45YCH6A Q45YCH6A | |  |
|  | **b.________________________________________________________________________________________________** | | | | Q45YCH6B Q45YCH6B | |  |
|  | **c.________________________________________________________________________________________________** | | | | Q45YCH6C Q45YCH6C | |  |
|  | **d._______________________________________________________________________________________________** | | | | Q45YCH6D Q45YCH6D | |  |

Now I would like you to just think about the past 12 months, that is since [refer to month one year ago]. Please tell me how often your child has had any of the following problems:

| 58 | In the past 12 months, how often has your child had a cold or flu? Would you say…. | Never……  Once or twice…….  Three or more times…. | 01  02  03 | Q46YCH7 Q46YCH7 |
| --- | --- | --- | --- | --- |
| 59 | How often has your child had malaria? Would you say………….. | Never……  Once or twice…….  Three or more times…. | 01  02  03 | Q47YCH8 Q47YCH8 |
| 60 | How often has your child had a headache? Would you say…. | Never……  Once or twice…….  Three or more times…. | 01  02  03 | Q48YCH9 Q48YCH9 |
| 61 | How often has your child had an upset stomach with vomiting, diarrhea or a fever? Would you say… | Never……  Once or twice…….  Three or more times…. | 01  02  03 | Q49YCH10 Q49YCH10 |
| 62 | Overall, would you say in general your child’s health is……….. | Poor…………………….  Fair……………………..  Good……………………  Excellent………………. | 01  02  03  04 | Q50YCH11 Q50YCH11 |

**Disabilities**

| 1. The next questions ask about your child’s behavior and feelings. I will read statements about common behaviors and feelings that most children have. Please give answers on the basis of how your child has been feeling over the last month. **(Put a X in the correct box for each question)** | **Not true**  **(1)** | **Somewhat True**  **(2)** | **Certainly True**  **(3)** | **Code** |
| --- | --- | --- | --- | --- |
| a. Tries to be nice to other people |  |  |  | Q61ASD1 Q61ASD1 |
| b. Considerate of other people’s feelings |  |  |  | Q61BSD2 Q61BSD2 |
| c. Restless, overactive, cannot sit still for long |  |  |  | Q61CSD3 Q61CSD3 |
| d. Often complains of headaches, stomach-aches or sickness |  |  |  | Q61DSD4 Q61DSD4 |
| e. Shares readily with other children (treats, toys, pencils etc.) |  |  |  | Q61ESD5 Q61ESD5 |
| f. Often has temper tantrums or hot tempers |  |  |  | Q61FSD6 Q61FSD6 |
| g. Rather solitary, tends to play alone |  |  |  | Q61GSD7 Q61GSD7 |
| h. Generally obedient, usually does what adults request |  |  |  | Q61HSD8 Q61HSD8 |
| i. Many worries, often seems worried |  |  |  | Q61ISD9 Q61ISD9 |
| j. Helpful if someone is hurt, upset or feeling ill |  |  |  | Q61JSD10 Q61JSD10 |
| k. Constantly fidgeting or squirming |  |  |  | Q61KSD11 Q61KSD11 |
| l. Has at least one good friend |  |  |  | Q61LSD12 Q61LSD12 |
| m. Often fights with other children or bullies them |  |  |  | Q61MSD13 Q61MSD13 |
| n. Can often make other people do what s/he wants |  |  |  | Q61NSD14 Q61NSD14 |
| o. Often unhappy, downhearted or tearful |  |  |  | Q61OSD15 Q61OSD15 |
| **Q61 cont.** | **Not true**  **(1)** | **Somewhat True**  **(2)** | **Certainly True**  **(3)** | **Code** |
| p. Generally liked by other children |  |  |  | Q61PSD16 Q61PSD16 |
| q. Easily distracted, concentration wanders |  |  |  | Q61QSD17 Q61QSD17 |
| r. Nervous or clingy in new situations, easily loses confidence |  |  |  | Q61RSD18 Q61RSD18 |
| s. Kind to younger children |  |  |  | Q61SSD19 Q61SSD19 |
| t. Often lies or cheats |  |  |  | Q61TSD20 Q61TSD20 |
| u. Picked on or bullied by other children |  |  |  | Q61USD21 Q61USD21 |
| v. Often volunteers to helps others (parents, teachers, other children) |  |  |  | Q61VSD22 Q61VSD22 |
| w. Thinks things out before acting |  |  |  | Q61WSD23 Q61WSD23 |
| x. Steals from home, school or elsewhere |  |  |  | Q61XSD24 Q61XSD24 |
| y. Gets on better with adults than with other children |  |  |  | Q61YSD25 Q61YSD25 |
| z. Many fears, easily scared |  |  |  | Q61ZSD26 Q61ZSD26 |
| aa. Sees tasks through to the end, good attention span |  |  |  | Q61AASD27 Q61AASD27 |

**Challenges of Raising a Teenager: Education**

| 64 | Is your child in school? | Yes (skip question 65)  No (go to question 65) | 01  02 | QN64CE |
| --- | --- | --- | --- | --- |
| 65 | What is the primary reason for not attending school? | Don’t know  Pregnancy  Parental Decision  Lack of School Contribution  Lack of uniform, school supplies  School full/ No place  Death of parent/guardian  Marriage  Adult in household sick/disabled  Child ill/disabled  Corporal punishment  Need to work  School too far  Other  Specify _________________ | 01  02  03  04  05  06  07  08  09  10  11  12  13  14 | QN65CE  QN65CEOT |
| 66 | Do you think your child will be able to finish primary school?  (skip if child is in secondary school) | Yes:  No:  Don’t know | 01  02  98 | Q62CE1 Q62CE1 |
|  |  | **Specify why Yes / No** ……………………………………………………………....... |  | Q62CE1Y Q62CE1Y |
| 67 | Would you like your child to finish secondary school? | Yes  No:  Don’t know | 01  02  98 | Q63CE2 Q63CE2 |
|  |  | **Specify why Yes / No** ……………………………………………………………… |  | Q63CE2Y Q63CE2Y |
| 68 | How important is it to you that your child does well in school? Would you say……… | Not at all important  Somewhat important  Very important | 01  02  03 | Q64CE3 Q64CE3 |
| 69 | How important is it to you that your child enjoys school? Would you say… | Not at all important  Somewhat important  Very important | 01  02  03 | Q65CE4 Q65CE4 |
| 70 | In the past week, how many times did you discuss school-related issues with your child? Would you say… | Never  Rarely (once a week)  Sometimes. (between 2-5 times a week)  All the time (more than 5 times a week) | 01  02  03  04 | Q66CE5 Q66CE5 |
| 71 | How does your child get to school in the morning? | Public bus or minivan  School bus  Car or taxi  Bicycle  Walk  Other (specify)  _____________ | 01  02  03  04  05  06 | QN71CE  QN71CEOT |
| 72 | Do you know the name of your child’s classroom teacher? | Yes  No | 01  02 | QN72CE |
| 73 | How has your child’s school performance/grades changed in the last year? | Improved  Declined  Stayed the Same  Don’t Know | 01  02  03  98 | QN73CE |
| 74a | Do you know your child’s grades on the Standard 4 exams? | Yes  No | 01  02 | Q74ACE |
| 74b | Do you know your child’s grades on the Standard 7 exams? | Yes  No | 01  02 | Q74BCE |

# Challenges of raising a teenager: Alcohol and Drugs

| 75. How often do you think your child has used any of the following in the last month? | Always  (1) | Often  (2) | Sometimes  (3) | Rarely  (4) | Never  (5) | DK  (6) | Code |
| --- | --- | --- | --- | --- | --- | --- | --- |
| a. Cigarettes |  |  |  |  |  |  | Q67ACA1 Q67ACA1 |
| b. Chewing tobacco or snuff |  |  |  |  |  |  | Q67BCA2 Q67BCA2 |
| c. Local brew, Beer, wine, hard liquors |  |  |  |  |  |  | Q67CCA3 Q67CCA3 |
| d. Marijuana (weed, grass, pot) |  |  |  |  |  |  | Q67DCA4 Q67DCA4 |
| e. Inhalants (glue, gas, paint, aerosols) |  |  |  |  |  |  | Q67ECA5 Q67ECA5 |
| f. Other (describe) |  |  |  |  |  |  | Q67FCA6 Q67FCA6SP  Q67FCA6S |

| 76 | How often do you discuss alcohol and drugs with your child? Would you say……………. | Never  A few times  Sometimes  Very frequently | 01  02  03  04 | Q68CA7: Q68CA7 |
| --- | --- | --- | --- | --- |
| 77 | How often does your child ask you questions about drugs and alcohol? Would you say…… | Never  Rarely (once a week)  Sometimes (between 2-5 times/week)  All the time (more than 5 times/week) | 01  02  03  04 | Q69CA8: Q69CA8 |

# Challenges of raising a teenager: Sex Education

| 78 | In general, how comfortable are you discussing sex education with your child? Would you say… | Not at all  Not really  Somewhat  Very | | 01  02  03  04 | Q70CSS1 Q70CSS1 |
| --- | --- | --- | --- | --- | --- |
| 79 | In general, do you think your child would approach you with questions about safe sex? Would you say… | Never  Sometimes  Most of the time  All the time | | 01  02  03  04 | Q71CSS2 Q71CSS2 |
| 80 | Do you feel you are aware of the changes going on in your child’s body (such as, for boys a deepening of the voice, for girls menstruation, for both a growth spurt)? Would you say……………. | I don’t know of any  I know of some changes  I am very aware of the changes | | 01  02  03 | Q72CSS3 Q72CSS3 |
| 81 | Whose responsibility is it to speak with your child about the changes in his/her body that occur during puberty? (new question) | mother only  father only  either parent  grandmother  grandfather  aunt  uncle  sister  brother  female/male friend  teacher  doctor/nurse  other  (specify)________ | | 01  02  03  04  05  06  07  08  09  10  11  12  13 | QN81CSS  QN81CSSO |
| 82 | In general, is it important for you to have conversations about safe sex with your child? Would you say… | Not at all important  Somewhat important  Very important | | 01  02  03 | Q73CSS4 Q73CSS4 |
| 83 | Has your child had any safe sex education in school, church/mosque, etc? | Yes (continue to Q84)  No (skip Q 84)  Don’t know | | 01  02  98 | Q74CSS5 Q74CSS5 |
| 84 | If yes, what was the content of the safe sex education?  ………………………………………………………………………………………………………………………… | | | | Q75CSS6 |
| 85 | Who do you believe is most responsible for talking to your child about sex? | Parents  Teacher  Religious leader  Other | | 01  02  03  04 | Q76CSS7 Q76CSS7 |
|  |  | **(specify)** ______________________ | |  | Q76CSS7S Q76CSS7SP |
| **86** | Have you discussed the following with your child?  a)Pregnancy (getting pregnant or making someone pregnant) | | Yes/No | 1 2 | Q77ACSS8 Q77ACSS8A |
|  | b)HIV/AIDS | | Yes/No | 1 2 | Q77BCSS8 Q77BCSS8B |
|  | c)Sexually transmitted diseases | | Yes/No | 1 2 | Q77CCSS8 Q77BCSS8C |

# Challenges of raising a teenager: Religious Faith

| **86** | In the last month, how often did your child go to religious services? | Never  1-3 times  4-6 times  More than 6 | 01  02  03  04 | Q78CRF1 Q78CRF1 |
| --- | --- | --- | --- | --- |
| 87 | Is your child involved in religious activities? | Yes  No  Don’t know | 01  02  98 | Q79CRF2 Q79CRF2 |
|  | If yes, specify activities  ……………………………………………………………………………………………………………………………….. | | | Q79CRF2Y Q79CRF2Y |

# Challenges of raising a teenager: Friendships

| 88 | How well do you feel you know your child’s friends? Would you say… | | I don’t know them at all  Not well  Kind of well  Very well | 01  02  03  04 | Q80CFR1: Q80CFR1 |
| --- | --- | --- | --- | --- | --- |
| 89 | How often do you talk about your child’s friends with your child? Would you say… | | Never  Rarely  Sometimes  All the time | 01  02  03  04 | Q81CFR2: Q81CFR2 |
| 90 | How often does your child seek your counsel about his/her friendships? Would you say… | Never  Rarely  Sometimes  All the time | | 01  02  03  04 | Q82CFR3: Q82CFR3 |
| 91 | How important is it to you to know about all your child’s relationships? | Not important at all  Somewhat important  Very important | | 01  02  03 | Q83CFR4: Q83CFR4 |

# Challenges of raising a teenager: Discipline

| 92 | In the past month about how many times have you physically punished [name]? Would you say…. | Not in last month  Once  A few times (2-5 times)  Many times (more than 5 ) | 01  02  03  04 | Q84CD1: Q84CD1 |
| --- | --- | --- | --- | --- |
|  | **We understand that all parents must discipline their child at some point. The next few questions ask about how you have disciplined your child over the last 12 months.** |  |  |  |
| 93 | What are methods you use to discipline a child the first time that s/he does something you believe to be wrong? | Reason with your child  Use restrictions  Hit with an object  Hit with your hand  Other  If Other Specify  ______________________ | 01  02  03  04  05 | QN93CD  QN93CDOT |
| 94 | If your child continues to disobey you, how do you discipline your child? | Reason with your child  Use restrictions  Hit with an object  Hit with your hand  Other  If Other Specify  ______________________ | 01  02  03  04  05 | QN94CD  QN94CDOT |
| 95 | In general, do you think that you get [name] to listen to you and do what you want him/her to do? Would you say… | Rarely  Some of the time  Most of the time | 01  02  03 | Q85CD2: Q85CD2 |
| 96 | In the past 30 days, how often do you think that your child has been involved in playing pool, card games or other games in which money is exchanged (gambling)? | Always  Often  Sometimes  Rarely  Never  Don’t Know | 01  02  03  04  05  98 | QN96CD |

# Challenges of raising a teenager: Technology

| 97 How many times do you think your child watched or used the following in the past 30 days? | Always  **(01)** | A Few Times  **(02)** | Sometimes  **(03)** | Rarely  **(04)** | Never  **(05)** | D/K  **(08)** |  |
| --- | --- | --- | --- | --- | --- | --- | --- |
| a. Television |  |  |  |  |  |  | Q86ACD3A Q86ACD3A |
| b. Video |  |  |  |  |  |  | Q86BCD3B Q86BCD3B |
| c. Internet |  |  |  |  |  |  | Q86CCD3C Q86CCD3C |
| d. Computer games |  |  |  |  |  |  | Q86DCD3D Q86DCD3D |
| e. Others (specify) |  |  |  |  |  |  | Q86ECD3E Q86ECD3ESP |

# Parental Efficacy

| 98 | How much could you do to influence your child’s academic future? | Nothing  Very little  Some  A lot | 01  02  03  04 | Q87CPE1: Q87CPE1 |
| --- | --- | --- | --- | --- |
| 99 | How much could you do to influence your child’s happiness? | Nothing  Very little  Some  A lot | 01  02  03  04 | Q88CPE2: Q88CPE2 |
| 100 | How much could you do to influence your child’s sense of security and belonging? | Nothing  Very little  Some  A lot | 01  02  03  04 | Q89CPE3: Q89CPE3 |
| 101 | How much could you do to influence your child’s sense of personal safety? | Nothing  Very little  Some  A lot | 01  02  03  04 | Q90CPE4: Q90CPE4 |
| 102 | How much could you do to influence your child’s healthy growth and development? | Nothing  Very little  Some  A lot | 01  02  03  04 | Q91CPE5: Q91CPE5 |
| 103 | How much could you do to influence your child to finish her /his homework? | Nothing  Very little  Some  A lot | 01  02  03  04 | Q92CPE6: Q92CPE6 |
| 104 | How much could you do to influence your child to use no alcohol? | Nothing  Very little  Some  A lot | 01  02  03  04 | Q93CPE7: Q93CPE7 |
| 105 | How much could you do to influence your child to choose friends who stay out of trouble? | Nothing  Very little  Some  A lot | 01  02  03  04 | Q94CPE8: Q94CPE8 |
| 106 | How much could you do to ensure your child feels safe approaching you with her /his personal problems? | Nothing  Very little  Some  A lot | 01  02  03  04 | Q95CPE9: Q95CPE9 |
| 107 | How much could you do to ensure your child is safe from sexual abuse by other family members? | Nothing  Very little  Some  A lot | 01  02  03  04 | Q96CPE10: Q96CPE10 |

CHILD ACTIVITIES

| 108 | Has your child worked in the past month? This includes all types of work, including farm work, paid work, and work for which your child is not paid. | Yes, for pay (cash or kind)  Yes, unpaid  No | 01  02  03 | QN108CAC |
| --- | --- | --- | --- | --- |
| 109 | How many days did your child work in the past month (both paid and unpaid)? | 1-7 days  8-14 days  15-20 days  Everyday  Does not work at all | 01  02  03  04  05 | QN109CAC |
| 110 | Does your child attend tuition classes outside the normal school hours? | Never  Once a week  3-4 times a week  Everyday | 02  03  04  01 | QN110CAC |
| 111 | Is your child a participant of any organized activities, such as Girl Guides, Boy Scouts or football teams? | Never  Once a week  3-4 times a week  Everyday | 02  03  04  01 | QN111CAC |

# CHILD SELF-EFFICACY

|  | Now that we have reflected on many different things, let’s think about the ways in which your child has changed in the last year.  We will be discussing many different areas in which your child may have changed in his/her confidence, please tell us if there were any changes. | | |  |
| --- | --- | --- | --- | --- |
| 112 | Over the last 12 months, how has your child changed in his/her ability to express his/her worries to you? | Improved  Declined  Stayed the same | 01  02  03 | QN112CHG |
|  | Comments: |  |  | QN112CHS |
| 113 | Over the last 12 months, how has your child’s ability to express his or herself created a change in how often your child is physically punished? | Punished more  Punished less  Stayed the Same | 01  02  03 | QN113CHG |
|  | Comments: |  |  | QN113CHS |
| 114 | Over the last 12 months, how has your child changed in his/her ability to express his or herself to peers? | Improved  Declined  Stayed the same | 01  02  03 | QN114CHG |
|  | Comments: |  |  | QN114CHS |
| 115 | Over the last 12 months, how has your child changed in his/her ability to express his or herself to teachers? | Improved  Declined  Stayed the same | 01  02  03 | QN115CHG |
|  | Comments: |  |  | QN115CHS |
| **116** | **Over the last 12 months, how has your child changed in his/her ability to express his or herself to other adults?** | **Improved**  **Declined**  **Stayed the same** | 01  02  03 | QN116CHG |
|  | Comments: |  |  | QN116CHS |
| 117 | Over the last 12 months, how has your child changed in his/her ability to express his or herself to siblings? | Improved  Declined  Stayed the same  Haimhusu | 01  02  03  04 | QN117CHG |
|  | Comments: |  |  | QN117CHS |

*Thank the participant for their cooperation*

**ASSESSMENT DEBRIEFING**

| 118 | Was the interview completed? | Yes  No | 1  2 | Q99BRPT Q99BRPT |  |
| --- | --- | --- | --- | --- | --- |
| 119 | If not, was this because the caregiver: | Could not interview  Refused  Other:  _____**___________** | 1  2  3 | Q100BRPT Q100BRPT  Q100BRPY |  |
| 120 | Did the caregiver understand most questions? | Yes  No | 1  2 | Q101BRPT Q101BRPT |  |
| 121 | How cooperative was the caregiver? | Very  Somewhat  Not cooperative | 1  2  3 | Q102BRPT Q102BRPT |  |
| 122 | Do you this the caregiver answered all sections honestly? | Yes  Some  No | 1  2  3 | Q103BRPT Q103BRPT |  |
| 123 | If no, which sections made you think this? | **(Write number of section(s) here)** |  | Q104BRPT Q104BRPT |  |
| 124 | Were there any problems during the interview because of the presence of others? | Yes  No | 1  2 | Q105BRPT Q105BRPT |  |
| 125 | What was the general mood of the interview? Comment: |  |  | QN125BRP |  |
| 126 | Did you follow sequencing of the questions as presented in the survey? | Yes  No  If No, Why? | 01  02 | QN126BRP  QN126BRN |  |

Other comments QN126BRX
