# Supplementary material for: Rural-urban disparities in the nutritional status of younger adolescents in Tanzania
Source: PLoS One. 2021 Dec 20;16(12):e0261480. doi: 10.1371/journal.pone.0261480 (PMC8687541; doi:10.1371/journal.pone.0261480)
Supplement: S2 File — (DOC) [file pone.0261480.s002.doc]

Moshi Child Health Survey

Harvard Medical School, Kilimanjaro Christian Medical Centre (KCMC) and National Bureau of Statistics (Tanzania)

August 2004

|  | Variable |  | Variable |
| --- | --- | --- | --- |
| Consent ID _ _ _ _ _ | CONSENTNO |  |  |
| Subject ID _ _ _ _ | RESPID | Date of Assessment  --/--/---- | DOQ |
| Interviewer ID - - | INTID | Date Edited by Int  --/--/---- | DOINT |
| Supervisor ID - - | SVRID | Date Edited by Sup  --/--/---- | DOSVR |
| Editor ID - - | EDID | Date Checked by Edi  --/--/---- | DOED |
| Data entry ID - - | DCLERK | Date entered  --/--/---- | DOE |
| Time began --.-- | TOB | Time ended --.-- | TOF |

*Adapted from Caribbean Youth Health Survey (Linda Halcon, University of Minnesota)*

Hello! My name is [interviewers name] and I work with Child Health and Social ecology project (CHASE in English)

#### **HISTORY**

You may remember that during the end of the year before last year, 2004, a researcher from CHASE asked you many questions about your health and your progress at home, at school, and neighborhood. Half of the number of children interviewed participated in Young Citizen Program (child to child) another half will begin the some program later this year. Today I would like to ask you the same questions you were asked the year before last year. A lot might have happened to you since the year before last year and we want to know if there any changes.

If you have already participated in the first Young Citizen program, please do not tell me. If I know that you have already participated, it might make me wish to hear some answers from you. This may also bring some difficulties for you to be honest during interview.

###### RESEARCH CONTENT

Let me tell you what will happen during the interview. I will repeat the same questions you were asked by CHASE researcher at the end of the year before last year, 2004. But, for now each child will be with a different researcher from the on in 2004, who does not know your answers for 2004 research, nor has he/she met you before and they don’t live in your neighborhood. These questions are not an exam. We just want to know more about you, how you progress with your studies, your physical health, your mental health, the feelings you have and how you confront them, and challenges you face as youth. There are also some few questions about malaria and HIV/AIDS

CONFIDENTIALITY

I will read to you some questions and fill the answers in this research form. Your name will not be written in the research. No one but only the head of research who is able to compare your name with your research answers. Results from this research might involve the community in order that other might be benefited from information we collected. Nevertheless, your name your name will not appear in the research results

**PaRTICIPATION**

During or after interview, it is possible that the superviser from CHASE team visits you again in order to confirm if you agreed and how interview is done. The supervisor may ask you again some of the questions that were asked at the beggining of interview.

We have talke to your parent/caregiver about what it means to be a participant in this project and you will be doing. He/she has given us permission for you to perticipate. Nevertheless that last dission about answering any question if from you. You may refuse to anser any question, for whatever reason. You may stop the interview at any time. No one will be angry or punish you for whaterver you decide.

Have you agreed to speak and should I ask you some question

Thank you

**NOW READ “STILL PICTURES AND VIDEO CONCENT FORM”**

**AND ASK IF A CHILD AGREES**

**FOR THIS CONCEnT YOU MUST GET A CHILD SIGNATURE**

| Section | Question number | Page |
| --- | --- | --- |
| 1. About you and your school |  |  |
| 3. Employment |  |  |
| 4. Tobacco, Alcohol, Drugs |  |  |
| 5. Sexuality |  |  |
| 6. Physical and Sexual Harm |  |  |
| 7. Behavior and Feelings |  |  |
| 8. Worries |  |  |
| 9. Your Family |  |  |
| 10. Relationships with others |  |  |
| 11. Your Health |  |  |
| 12. Medical Care |  |  |
| 13. Malaria |  |  |
| 14. Pubertal development and nutrition | 83-92 | 22-24 |
| 15. Self-efficacy | 93-97 | 24-25 |

**ABOUT YOU AND SCHOOL**

These are some general questions about you, what you like to do, and how you

feel about school.

|  | | Question | | | |  | | Variable Name  HA-2 HA-1 | | | | | | |  | | |
| --- | --- | --- | --- | --- | --- | --- | --- | --- | --- | --- | --- | --- | --- | --- | --- | --- | --- |
| 1 | | What is the name of your ward? | | | | ………………… | | Q1YSCH --------- | | | | | | |  | | |
| 2a | | What is the name of your mtaa | | | | ………………… | | Q2AYSCH Q2YSCH | | | | | | |  | | |
| 2b | | What is the name of your mtaa leader? (new question) | | | |  | | Q2NBYSCH | | | | | | |  | | |
| 3a  3b | | What is the name of your school?  In which Ward? | | | | …………………  ………………… | | Q3AYSCH Q3AYSCH  Q3BYSCH Q3BYSCH | | | | | | |  | | |
| 3c | Is your school: | | | | | | Public  Private  Mission | 01  02  03 | | Q3CYSCH Q3CYSCH | | | | | |  | |
| 4a | What is your date of birth?  (**If don’t know: 98/98/2098)** | | | | | | __/__/__  **day/mth/yr** |  | | Q5AYSCH Q5AYSCH | | | | | |  | |
| 4b | How old are you now? | | | | | | **(Write age in years)** |  | | Q5BYSCH Q5BYSCH | | | | | |  | |
| 5 | What is your sex? | | | | | | Male  Female | 01  02 | | Q6YSCH Q6YSCH | | | | | |  | |
| 6a | Have you ever attended school? | | | | | | Yes  No **(skip to question 19)** | 01  02 | | Q8AYSCH Q8YSCH | | | | | |  | |
| 6B | What grade are you in now? | | | | I am not in school **🡪**  **(Skip to question 19)**  Standard one  Standard two  Standard three  Standard four  Standard five  Standard six  Standard seven  Form I  Form II  Form III  Vocational Technical School  Other (specify) ____________________ | | | 00  01  02  03  04  05  06  07  08  09  10  11  12 | | Q8CYSCH Q8CYSCH  Q8CYSCHO Q8CYSCHOT | | | | | |  | |
| 7a | What kind of vocational training are you receiving?  (new question) | | | | Vocational Training School  Temporary attendance to gain some technical knowledge | | | 01  02 | | QN7AYSCH | | | | | |  | |
| 7b | Are you a part-time or full-time student? (new question) | | | | Full-time  Part-time | | | 01  02 | | QN7BYSCH | | | | | |  | |
| 8 | How long does it take for you to get to school in the morning? | | | | Less than half an hour  Half an hour to an hour  More than one hour | | | 01  02  03 | | Q9YSCH Q9YSCH | | | | | |  | |
| 9 | How do you get to school?  **(Mark all that apply)**  1=Ndiyo 2=Hapana | | A. Public Bus or mini-van (Hiace)  B. School Bus  C. Car or taxi  D. Bicycle  E. Walk  F. Other (EXPLAIN)  (new response) | | | | | | 1 2  1 2  1 2  1 2  1 2  1 2 | | | | | Q10ASCH Q10AYSCH  Q10BYSCH Q10BYSCH  Q10CYSCH Q10CYSCH  Q10DYSCH Q10DYSCH  Q10EYSCH Q10EYSCH  Q10FYSCH |  | | |
| 10 | What do you like best about your school?  **(Choose one option)** | | | My teachers  Reading Swahili stories  Learning English  Playing with friends  Sports sessions  Getting away from work at home  I like the food/ I having enough lunch  Other: **(Specify)** ______________________ | | | | 01  02  03  04  05  06  0708 | | Q11YSCH Q11YSCH  Q11YSCHO Q11YSCHOT | | | | | |  | |
| 11 | What do you like least about your school?  **(Choose one option)** | | | My teachers  Transport to and from school  School work  Corporal punishment (canning, physical)  Outside classroom work  Aggressive kids  Not having friends  I do not like the food/not enough to eat  Other **(specify):** ______________________ | | | | 01  02  03  04  05  06  07  08  09 | | Q12YSCH Q12YSCH  Q12YSCHO Q12YSCHOT | | | | | |  | |
| 12 | Would you like to finish primary school? | | | | Yes  No **(specify why)**  ____________________  Don’t know | | | 01  02  98 | | Q13YSCH Q13YSCH  Q13YSCHN Q13YSCHNO | | | | | |  | |
| 13 | Would you like to finish secondary school? | | | | Yes  No **(specify why)**  ____________________  Don’t know | | | 01  02  98 | | Q14YSCH Q14YSCH  Q14YSCHN Q14YSCHNO | | | | | |  | |
| 14 | Does your classroom teacher know your name? | | | | Yes  No | | | 01  02 | | Q15YSCH Q15YSCH | | | | | |  | |
| 15 | Do you have trouble getting your homework done? | | | | Always  Never  Sometimes | | | 01  02  03 | | Q16YSCH Q16YSCH | | | | | |  | |
| 16A | Is keeping up with your schoolwork hard because you have trouble reading school books (in Swahili)? | | | | Yes  No  Sometimes | | | 01  02  03 | | Q17YSCH Q17YSCH | | | | | |  | |
| 16B | In the past 12 months, how has your school performance/grades changed?  (Read the choices)  (new question) | | | | Improved  Declined  Stayed the same | | | 01  02  03 | | Q16BYSCH | | | | | |  | |
| 17 | How often were you caned in school in the past month?  **(Now skip to Q21)** | | | | Never  1-3 times  4-6 times  6-8 times  More than 8 times | | | 01  02  03  04  05 | | Q18YSCH Q18YSCH | | | | | |  | |
|  | **(If child has never attended school/is not currently in school please ask questions 19-20)** | | | | | | | | | |  |  |  | | | | |
| 18 | What is the primary reason for not attending school?  **(Do not read out these options; check all that apply)** | | | **A)**Don’t know  **B)**Pregnancy  **C)**Parental decision  **D)**Lack of School Contribution  **E)**Lack of uniform, school supplies  **F)**School full/no place  **G)**Death of parent/guardian  **H)**Early marriage  **I)**Adult in household sick/disabled  **J)**I was ill/disabled  **K)**Corporal punishment  **L)**Need to work  **M)**School too far  **N)**Other  **(specify)**  _________________________ | | | | | 1 2  1 2  1 2  1 2  1 2  1 2  1 2  1 2  1 2  1 2  1 2  1 2  1 2  1 2 | | | | | Q19AYSCH Q19AYSCH  Q19BYSCH Q19BYSCH  Q19CYSCH Q19CYSCH  Q19DYSCH Q19DYSCH  Q19EYSCH Q19EYSCH  Q19FYSCH Q19FYSCH  Q19GYSCH Q19GYSCH  Q19HYSCH Q19HYSCH  Q19IYSCH Q19ISCH  Q19JYSCH Q19JYSCH  Q19KYSCH Q19KYSCH  Q19LYSCH Q19LYSCH  Q19MYSCH Q19MYSCH  Q19NYSCH Q19NYSCH  Q19YSCHS |  | | |
| 19 | Would you like to go to school? | | | | Yes  No **(specify reason)**  ____________________  I don’t know | | | 01  02  98 | | Q20YSCH1 Q20YSCH  Q20YSCH2 Q20YSCHNO | | | | | |  | |
| 20 | **(If dropped out of school)**  What was the last grade you completed? | | | | Pre Standard one  Standard one  Standard two  Standard three  Standard four  Standard five  Standard six  Standard seven  Form I  Form II  Form III  Other (specify)  ____________________ | | | 00  01  02  03  04  05  06  07  09  10  11  08 | | Q20AYSCH Q20AYSCH  Q20YASCO Q20AYSCHOT | | | | | | |  |

**ALL RESPONDENTS**

| 21a | Do you participate in any organized activities such as girl guides, boy scouts or sports teams? | Every day  Never  Once a week  3-4 times a week |  | Q21AYSCH Q21AYSCH |
| --- | --- | --- | --- | --- |
| 21b | Do you attend tuition classes outside the normal school hours? | Every day  Never  Once a week  3-4 times a week |  | Q21BYSCH Q21BYSCH |
| 22 | How often in the past 30 days have you played cards, pool or other games where money is exchanged (gambling)?  (new question) | Always  Often  Sometimes  Rarely  Never | 01  02  03  04  05 | QN22YSCH |

Employment

| 23 | Have you worked in the past month? This includes all types of work, including farm-work, paid work and work that you do not get paid for. | | Yes, at least once I worked for pay (cash or kind)  Yes, unpaid  No **-> skip to 30.** | 01  02  03 | Q25EMP Q25EMP |
| --- | --- | --- | --- | --- | --- |
| 24 | How many days did you work in the past month (both paid and unpaid)? | 1-7 days  8-14 days  15-20 days  Every day  Does not work at all | | 01  02  03  04  05 | Q27EMP Q27EMP |
| 25 | How many months did you work in the last year (both paid and unpaid)? | | 0 months  1-3 months  4-6 months  7-9 months  10-12 months | 01  02  03  04  05 | Q28EMP Q28EMP |
| 26 | During the school year, how many hours per week do you work for pay? | | 1-4 hours a week  5-9 hours a week  10-20 hours a week  Over 20 hours a week  Does not work for pay | 01  02  03  04  05 | Q29EMP Q29EMP |
|  |  | |  |  |  |

**TOBACCO, ALCOHOL, AND OTHER SUBSTANCES**

The use of alcohol and drugs is a major issue for many youths. Please help us understand this issue by answering the following questions honestly and completely. Remember, your answers will be kept secret.

| 27 How often have you used the following things during the last month? | | | | | |  |  | |  | |
| --- | --- | --- | --- | --- | --- | --- | --- | --- | --- | --- |
|  | Never  **(1)** | Rarely  (1/2 times)  **(2)** | Sometimes  **(3)** | Often  **(4)** | Always  **(5)** | | | DK  **(6)** |  |  |
| a. Cigarettes |  |  |  |  |  | | |  | Q30ATXC | Q30ATXC |
| b. Chewing tobacco or snuff, *mirungi* |  |  |  |  |  | | |  | Q30BTXC | Q30BTXC |
| c. Local brew, beer |  |  |  |  |  | | |  | Q30CTXC | Q30CTXC |
| d. Imported beer, wine, spirits |  |  |  |  |  | | |  | Q30DTXC | Q30DTXC |
| e. Marijuana (weed, grass, pot) |  |  |  |  |  | | |  | Q30ETXC | Q30ETXC |
| f. Inhalants (glue, gas, paint, aerosols) |  |  |  |  |  | | |  | Q30FTXC | Q30FTXC |
| g. Other (describe)  _______________________ |  |  |  |  |  | | |  | Q30GTXCS | Q30GTXC  Q30GTXCSP |

**SEXUALITY**

Sex is an important part of people’s lives. Though it is very private, we hope that you will share some information with us so we can better understand the concerns and questions of people your age. Remember that your answers will be kept private.

| 28 | Do you know if any of your friends have had any kind of sexual experiences? | Yes  No  Don’t know | 01  02  98 | Q31SXL Q31SXL |
| --- | --- | --- | --- | --- |
| 29 A | Have you ever had any kind of sexual experience (such as kissing, petting) | Yes  No | 01  02 | Q32SXL Q32SXL |
| 29B | In the last 12 months have you had any kind of sexual experience (such as kissing or petting) (new question) | Yes  No | 01  02 | QN29BSXL |
| 30A | Have you ever had sexual  Intercourse? | Yes  No | 01  02 | Q33SXL Q33SXL |
| 30B | In the last 12 months have you had sexual intercourse?  (new question) | Yes, because **(go to question # 32a)**  No, because  **(mark all that apply in Q32b then skip to question #38)** | 01  02 | QN30BSXL |
| 31a | Yes, give reasons:  Temptations (money, gift, etc)  Forced/threatened  Parents sending for family needs/supplies (without giving money)  (imitating/watching) Video  Drugs/alcohol  Poor/Bad upbringing  Peer group pressure  Wanting to try  Other (explain/state)  (new question) | | 01  02  03  04  05  06  07  08 | QN31ASXL  QN31ASXO |
| 31B | No, give reasons:  Wants to wait until older  Wants to wait until married  Not emotionally ready for it  Don’t want to risk becoming pregnant/ getting someone pregnant  Haven’t met anyone wants to have sex with me  Haven’t had the opportunity to have sex with someone he/she like  Fears disease (AIDS, other STDs)  Religious values are against it  Parent’s values are against it  Wants to, but no one has asked me  Would feel guilty  Just don’t feel like it (don’t feel the need to have sex) | | 01  02  03  04  05  06  07  08  09  10  11  12 | Q33ASXL Q33ASXL |
| 32 | The first time you had intercourse, were you forced into it against your will? | Yes  No  Sort of | 01  02  03 | Q34SXL Q34SXL |
| 33 | How old were you the first time you had sexual intercourse? | Write the age in years  **(prompt: Younger than 10? This year? Last year?)** |  | Q35SXL Q35SXL |
| 34a | How old was your partner | 10 years old or younger  11 years old  12 years old  13 years old  14 years old  15 to 18 years  Older than 18 years | 01  02  03  04  05  06  07 | Q36SXL Q36SXL |
| 34b | Who was your partner?  (new question) | Someone I know  Someone I don’t know  Boyfriend/Girlfriend  Other | 01  02  03  04 | QN35BSXL  QN35BSXO |
| 35 | How many people have you had sex with during your life? | 1 person  2-3 people  4-5 people  6-10 people  more than 10 people | 01  02  03  04  05 | Q37SXL Q37SXL |
| 36 | **(If female)** Have you ever been pregnant?  **(If male)** Have you ever made someone pregnant? | Yes  No  Don’t know  Refused | 01  02  98  99 | Q38SXLA  (if female) Q38SXL  Q38SXLB  (if male) |

ALL RESPONDENTS

| 37 | At what age do you think it is all right for a person to get pregnant? | 11 years old or younger  12 years old  13 years old  14 years old  15 years old  16 years old  17 years old  18 years old  19 years old  20 years old  Over 20 years old | 01  02  03  04  05  06  07  08  09  10  11 | Q39SXL Q39SXL |
| --- | --- | --- | --- | --- |

**PHYSICAL AND SEXUAL HARM**

| 38a | Have you ever been physically harmed or mistreated by anyone in your family or anyone else? Physical harm is when someone causes you to have a scar, black and blue marks, welts, bleeding, or a broken bone. | | Yes  No  Sort of | | 01  02  03 | Q51PSA Q51PSA |
| --- | --- | --- | --- | --- | --- | --- |
| 38b | **If “Sort of”, or “Yes” ask:**  Who was is?  **(Mark all that apply)** | **A)**Refused  **B)**A parent  **C)**A relative who lives with me  **D)**A relative who does not live with me  **E)**A brother, sister or other teenager who lives with me  **F)** A boyfriend, girlfriend or other teenager who does NOT live with me  **G)** A teacher  **H)** A priest, minister, sheik or other religious leader  **I)** Any other adult | | 1 2  1 2  1 2  1 2  1 2  1 2  1 2  1 2  1 2 | | Q51APSA Q51APSA  Q51BPSA Q51BPSA  Q51CPSA Q51CPSA  Q51DPSA Q51DPSA  Q51EPSA Q51EPSA  Q51FPSA Q51FPSA  Q51GPSA Q51GPSA  Q51HPSA Q51HPSA  Q51IPSA Q51IPSA |
| 39a | Have you ever been sexually abused? Sexual harm is when someone in your family or someone else touches you in a place you did not want to be touched, or does something sexually which they shouldn’t have done to you, or forces you to touch them sexually or have sex with them. | | Yes  No  Sort of | | 01  02  03 | Q52PSA Q52PSA |
| 39b | **If “Sort of”, or “Yes” ask:**  Who was is?  **(Mark all that apply)** | **A)** Refused  **B)** A parent  **C)** A relative who lives with me  **D)** A relative who does not live with me  **E)** A brother, sister or other teenager who lives with me  **F)** A boyfriend, girlfriend or other teenager who does NOT live with me  **G)** A teacher  **H)** A priest, minister, sheik or other religious leader  **I)** Any other adult | | 1 2  1 2  1 2  1 2  1 2  1 2  1 2  1 2  1 2 | | Q52APSA Q52APSA  Q52BPSA Q52BPSA  Q52CPSA Q52CPSA  Q52DPSA Q52DPSA  Q52EPSA Q52EPSA  Q52FPSA Q52FPSA  Q52GPSA Q52GPSA    Q52HPSA Q52HPSA  Q52IPSA Q52IPSA |

**BEHAVIOR AND FEELINGS**

40. The next questions ask about your behavior and feelings. I will now read statements about common behaviors and feelings that most youths have. For each of the statements put a tick in the box that you think is most like you. Please give answers on the basis of how you have been feeling over the last month.

|  | Not true  **(1)** | Somewhat true  **(2)** | Certainly True  **(3)** |  |  |
| --- | --- | --- | --- | --- | --- |
| a. You try to be nice to people. |  |  |  | Q53ABF | Q53ABF |
| b. You care about their feelings. |  |  |  | Q53BBF | Q53BBF |
| c. You get restless; You cannot sit still for long. |  |  |  | Q53CBF | Q53CBF |
| d. You get a lot of headaches, stomachaches or are often sick. |  |  |  | Q53DBF | Q53DBF |
| e. You usually share with others (food, games, pens etc.) |  |  |  | Q53EBF | Q53EBF |
| f. You get very angry and often lose your temper. |  |  |  | Q53FBF | Q53FBF |
| g. You are usually on your own. You generally play alone or keep to yourself. |  |  |  | Q53GBF | Q53GBF |
| h. You usually do as you are told. |  |  |  | Q53HBF | Q53HBF |
| i. You worry a lot. |  |  |  | Q53IBF | Q53IBF |
| j. You are helpful if someone is hurt, upset or feeling ill. |  |  |  | Q53JBF | Q53JBF |
| k. You are constantly fidgeting or squirming. |  |  |  | Q53KBF | Q53KBF |
| l. You have one good friend or more. |  |  |  | Q53LBF | Q53LBF |
| m. You fight a lot. |  |  |  | Q53MBF | Q53MBF |
| n. You can make other people do what you want. |  |  |  | Q53NBF | Q53NBF |
| o. You are often unhappy, downhearted or tearful. |  |  |  | Q53OBF | Q53OBF |
| p. Other people your age generally like you. |  |  |  | Q53PBF | Q53PBF |
| q. You are easily distracted. You find it difficult to concentrate. |  |  |  | Q53QBF | Q53QBF |
| r. You are nervous in new situations. You easily lose confidence. |  |  |  | Q53RBF | Q53RBF |
| s. You are kind to younger children. |  |  |  | Q53SBF | Q53SBF |
| t. You are often accused of cheating or lying. |  |  |  | Q53TBF | Q53TBF |
| u. Other children or young people pick on or bully you. |  |  |  | Q53UBF | Q53UBF |
| v. You often volunteer to help others (parents, teachers, children.) |  |  |  | Q53VBF | Q53VBF |
| w. You think before you do things. |  |  |  | Q53WBF | Q53WBF |
| x. You take things that are not yours from home, school or elsewhere. |  |  |  | Q53XBF | Q53XBF |
| y. You get on better with adults than with people your own age. |  |  |  | Q53YBF | Q53YBF |
| z. You have many fears. You are easily scared. |  |  |  | Q53ZBF | Q53ZBF |
| aa. You finish the things you are doing. Your concentration is good. |  |  |  | Q53AABF | Q53AABF |

41. How often have you done the following during the past month?

|  | Never  **(1)** | Once or twice  **(2)** | Three or more  **(3)** |  |  |
| --- | --- | --- | --- | --- | --- |
| a. Skipped school without an excuse |  |  |  | Q54ABF | Q54ABF |
| b. Cheated on a test |  |  |  | Q54BBF | Q54BBF |
| c. Deliberately damaged property |  |  |  | Q54CBF | Q54CBF |
| d. Been in a fight where weapons were used(sticks, stones, knives,razors) |  |  |  | Q54DBF | Q54DBF |
| e. Took something from a store, shop, or supermarket without paying for it |  |  |  | Q54EBF | Q54EBF |
| f. Stole something from your parents or other family members |  |  |  | Q54FBF | Q54FBF |
| g. Went into a house or building to steal something |  |  |  | Q54GBF | Q54GBF |
| h. Run away from home |  |  |  | Q54HBF | Q54HBF |

**WORRIES**

42. The next questions ask you how much you worry about different things that might

happen in your life. For each thing, tell me how much you worry about it.

| 44. You worry about… | Not at all  **(1)** | Some  **(2)** | A lot  **(3)** | Not r. vant **(4)** |  |  |
| --- | --- | --- | --- | --- | --- | --- |
| a. Missing a meal |  |  |  |  | Q55AWR | Q55AWR |
| b. Your own drinking |  |  |  |  | Q55BWR | Q55BWR |
| c. Your own drug use |  |  |  |  | Q55CWR | Q55CWR |
| d. Your mother or father’s drinking |  |  |  |  | Q55DWR | Q55DWR |
| e. Your mother or father’s using drugs |  |  |  |  | Q55EWR | Q55EWR |
| f. Being punished by parents |  |  |  |  | Q55FWR | Q55FWR |
| g. Being punished by teachers |  |  |  |  | Q55GWR | Q55GWR |
| h. Being sexually harmed |  |  |  |  | Q55HWR | Q55HWR |
| i. All the fighting and violence you see in your home |  |  |  |  | Q55IWR | Q55IWR |
| j. The violence in your community |  |  |  |  | Q55JWR | Q55JWR |
| k. The drinking in your neighborhood |  |  |  |  | Q55KWR | Q55KWR |
| l. The drug use in your neighborhood |  |  |  |  | Q55LWR | Q55LWR |
| m. Getting or making someone pregnant |  |  |  |  | Q55MWR | Q55MWR |
| n. Getting AIDS |  |  |  |  | Q55NWR | Q55NWR |
| o. Having many deaths in your family |  |  |  |  | Q55OWR | Q55OWR |
| p. Being an orphan |  |  |  |  | Q55PWR | Q55PWR |
| q. Getting a job when you are older |  |  |  |  | Q55QWR | Q55QWR |
| r. Finishing primary school |  |  |  |  | Q55RWR | Q55RWR |
| s. Passing Common Entrance/CXC the National Exam (Standard 7) |  |  |  |  | Q55SWR | Q55SWR |
| t. Getting a chance to attend secondary school |  |  |  |  | Q55TWR | Q55TWR |
| u. Feeling safe at school |  |  |  |  | Q55UWR | Q55UWR |
| v. Having basic needs at school (lunch, uniform, books) |  |  |  |  | Q55VWR | Q55VWR |
| w. Having basic needs at home (food, clothes, shelter) |  |  |  |  | Q55WWR | Q55WWR |

| 43 | Overall, do you see yourself as a person who is…?  (**read each option)** | Happy  Sad  Angry  Irritable | 01  02  03  04 | Q56WR Q56WR |
| --- | --- | --- | --- | --- |
| 44 | Please state why you feel this way **(in Q 56)** | __________________________  __________________________ |  | Q57WR Q57WR |
| 45 | If you had to move from this neighborhood to another neighborhood in Moshi,  how happy or unhappy would you be……? | Unhappy  It would make no difference  Happy | 01  02  03 | Q58WR Q58WR |

**YOUR FAMILY**

| 46 | Where do you usually sleep? | | In your home  At other people’s homes (friend, relative)  On the street  Orphanage or children’s center  Guest house or hostel  I have more than one usual sleeping place | | 01  02  03  04  05  06 | Q59FML Q59FML |
| --- | --- | --- | --- | --- | --- | --- |
| 47 | How many people slept there last night? | | 1 (lives alone)  2  3  4  5  6  7  8  9 or more | | 01  02  03  04  05  06  07  08  09 | Q60FML Q60FML |
| 48 | Is the number of people who slept there last night | | Less than usual  The usual number  More than usual | | 01  02  03 | Q61FML Q61FML |
| 49 | How many other people sleep in the same room with you? | | Sleeps alone  1 other person  2 other people  3-4 others  5-6 others  7-8 others  9 or more others | | 01  02  03  04  05  06  07 | Q62FML Q62FML |
| 50A | Is your mother alive? | | Yes  No  Don’t know | | 01  02  98 | Q63FML Q63FML |
| 50b | **(If mother dead)** Year of death | | __ __ __ __ | |  | Q63AFML Q63AFML |
| 51 | Is your father alive? | | Yes  No  Don’t know | | 01  02  98 | Q64FML Q64FML |
| 51a | **(If father dead)** Year of death | | __ __ __ __ | |  | Q64AFML Q64AFML |
| 52 | **(If both parents are alive)** Are your parents……? | | Living together  Living separately or apart  Don’t know | | 01  02  98 | Q65FML Q65FML |
| 53 | With whom do you spend MOST of your time? (**Mark all that are true for you**.) | **A)**Lives alone  **B)**Two parents (birth parents, adoptive parents and/or step parents)  **C)**One parent – mother only  **D)**One parent – father only  **E)**House Servant (Ayah)  **F)**Other adult relative (grandparents, aunt, uncle)  **G)**Other adult not related to you (foster parent, step parent, friend, etc.)  **H)**Other youth (brothers, sisters, step brother, step sisters, friends, etc.) | | 1 2  1 2  1 2  1 2  1 2  1 2  1 2  1 2 | | Q66AFML Q66AFML  Q66BFML Q66BFML  Q66CFML Q66CFML  Q66DFML Q66DFML  Q66EFML Q66EFML  Q66FFML Q66FFML  Q66GFML Q66GFML  Q66HFML Q66HFML |
| 54 | Which adult are you closest to? | | Mother  Father  Grandparent, aunt, uncle  Other **(specify)**  _____________________________ | | 01  02  03  04 | Q67FML Q67FML  Q67FMLSP |
| 55 | In the past month, how often did you go to religious services? | | Never  1-3 times  4-6 times  More than 6 times | | 01  02  03  04 | Q69FML Q69FML |
|  |  | |  | |  |  |

**RELATIONSHIPS WITH OTHERS**

These are some questions about your feelings and your relationships with others.

| 56. How much do you feel…? | Does not apply to me  **(1)** | Very little  **(2)** | Somewhat  **(3)** | A lot  **(4)** |  |  |
| --- | --- | --- | --- | --- | --- | --- |
| a. Your mom cares about you |  |  |  |  | Q70ARLT | Q70ARLT |
| b. Your dad cares about you |  |  |  |  | Q70BRLT | Q70BRLT |
| c. Other family members care about you |  |  |  |  | Q70CRLT | Q70CRLT |
| d. Adults in the neighborhood care about you |  |  |  |  | Q70DRLT | Q70DRLT |
| e. Your friends care about you |  |  |  |  | Q70ERLT | Q70ERLT |
| f. Your teachers care about you |  |  |  |  | Q70FRLT | Q70FRLY |
| g. Your priest, minister, sheik cares about you |  |  |  |  | Q70GRLT | Q70GRLT |

| 57. How much do you feel…? | | Does not apply to me  **(1)** | Very little  **(2)** | | Somewhat  **(3)** | A lot  **(4)** | |  |  |
| --- | --- | --- | --- | --- | --- | --- | --- | --- | --- |
| a. You can tell you mom about your problems | |  |  | |  |  | | Q71ARLT | Q71ARLT |
| b. You can tell your dad about your problems | |  |  | |  |  | | Q71BRLT | Q71BRLT |
| c. Your family protects you | |  |  | |  |  | | Q71CRLT | Q71CRLT |
| d. You want to run away from home | |  |  | |  |  | | Q71DRLT | Q71DRLT |
| 58a | In the past 12 months how often do you help out around the house (like cleaning the floor, sweeping, washing, etc) without being asked? (new question) | | | Everyday  Never  Sometimes  N/A | | | QN58ARLT | | |
| 58B | We know that often parents/guardians use different methods of punishing children when they do a wrong to them/have made a mistake.  The first time that you do something your parents believe to be wrong, how do they discipline you? (new question) | | | Talk with me (forgive me, give me a warning)  Prevent me from doing what I enjoy (give me work)  Hit with Object (whip)  Hit with Hand  Other | | | QN58BRLT  QN58BRTO | | |
| 58C | If you continue to disobey, how do your parents discipline you then? (new question) | | | Talk with me (forgive me, give me a warning)  Prevent me from doing what I enjoy (give me work)  Hit with Object (whip)  Hit with Hand  Other | | | QN58CRLT  QN58CRTO | | |
| 58D | If you were physically abused, to whom would you first turn? (new question) | | | Mother  Father  Grandmother  Grandfather  Other relative  Teacher  Mtaa leader  Doctor/Nurse  Counselor (KWEICO/KIWAKUKKI)  Other person  (specify) | | | QN58DRLL  QN58DRL2  QN58DRL3  QN58DRL4  QN58DRL5  QN58DRL6  QN58DRL7  QN58DRL8  QN58DRL9  QN58DR10  QN58D10O | | |
| 58e | Who else would you go and see? (new question) | | | Mother  Father  Grandmother  Grandfather  Other relative  Teacher  Mtaa leader  Doctor/Nurse  Counselor (KWEICO/KIWAKUKKI)  Other person | | | QN58ERL1  QN58ERL2  QN58ERL3  QN58ERL4  QN58ERL5  QN58ERL6  QN58ERL7  QN58ERL8  QN58ERL9  QN58ERL0  QN58ERLT | | |
| 58f | If you were sexually abused, to whom would you first turn? (new question) | | | Mother  Father  Grandmother  Grandfather  Other relative  Teacher  Mtaa leader  Doctor/Nurse  Counselor (KWEICO/KIWAKUKKI)  Other person | | | QN58FRL1  QN58FRL2  QN58FRL3  QN58FRL4  QN58FRL5  QN58FRL6  QN58FRL7  QN58FRL8  QN58FRL9  QN58FRL0  QN58FRLT | | |
| 58g | Who else would you go and see? (new question) | | | Mother  Father  Grandmother  Grandfather  Other relative  Teacher  Mtaa leader  Doctor/Nurse  Counselor (KWEICO/KIWAKUKKI)  Other person | | | QN58GRL1  QN58GRL2  QN58QRL3  QN58GRL4  QN58GRL5  QN58GRL6  QN58GRL7  QN58GRL8  QN58GRL9  QN58GRL0  QN58GRLT | | |

GENERAL HEALTH

| 59 | In general, how is your health? | Poor  Fair  Good  Excellent | 01  02  03  04 | Q72HLT Q72HLT |
| --- | --- | --- | --- | --- |
| 60 | Do you have a condition (handicap, disability, chronic illness) that limits you doing the same things other people your age do such as school sports, getting together with friends? | Yes  No  If Yes, describe:  ______________________  ______________________ | 01  02 | Q73HLT Q73HLT  Q73HLTY Q73HLTY |
| 61 | How many times per week do you work, play or exercise hard enough to make you sweat and breathe heavily? | Never  1 or 2 times  3 to 5 times  6 or more times | 01  02  03  04 | Q74HLT Q74HLT |

62. Do any of the following cause you regular problems (more than once or twice a week)?

|  | Hardly Ever  **(1)** | Sometimes  **(2)** | A lot  **(3)** |  |  |
| --- | --- | --- | --- | --- | --- |
| a. Headaches |  |  |  | Q75AHLT | Q75AHLT |
| b. Toothaches |  |  |  | Q75BHLT | Q75BHLT |
| c. Skin problems |  |  |  | Q75CHLT | Q75CHLT |
| d. Trouble seeing (even with glasses) |  |  |  | Q75DHLT | Q75DHLT |
| e. Trouble hearing |  |  |  | Q75EHLT | Q75EHLT |
| f. Stomach aches |  |  |  | Q75FHLT | Q75FHLT |
| g. Trouble breathing |  |  |  | Q75GHLT | Q75GHLT |
| h. A heart problem |  |  |  | Q75HHLT | Q75HHLT |
| i. Not getting enough sleep |  |  |  | Q75IHLT | Q75IHLT |
| j. Difficulty using hands, arms, legs or feet |  |  |  | Q75JHLT | Q75JHLT |
| k. Gets malaria easily |  |  |  | Q75KHLT | Q75KHLT |
| l. Coughs a lot |  |  |  | Q75LHLT | Q75LHLT |
| m. Faints (pass out sometimes) |  |  |  | Q75MHLT | Q75MHLT |
| n. Gets diarrhea often |  |  |  | Q75NHLT | Q75NHLT |
| o. To feel very tired (new question) |  |  |  | Q75OHLT |  |
| p. Blood in the urine (new question) |  |  |  | Q75PHLT |  |
| q. Ulcers/Sores (new question) |  |  |  | Q75QHLT |  |
| 63. How many days during the past 30 days you could not go to/be in school due to physical or mental health problems? (new question) | | | Days ___ | QN63HLT |  |

MEDICAL CARE

| 64 | Where do you usually go for medical care?  (**Mark all that apply)** | **A)** Nowhere  **B)** Public Clinic (hospital, health center, health post, dispensary)  **C)** Private Clinic (hospital, health center, health post, dispensary)  **D)** Traditional healer, herbalist, bush doctor | 1 2  1 2  1 2  1 2 | Q76ATRT Q76ATRT  Q76BTRT Q76BTRT  Q76CTRT Q76CTRT  Q76DTRT Q76DTRT |
| --- | --- | --- | --- | --- |
| 65 | Do you know the ways that pregnancy can be prevented? **(Unprompted; mark all that are mentioned)** | **A)** Don’t know  **B)** Abstinence  **C)** Birth Control Pills  **D)** Depo-Provera  **E)** Condoms (male and female)  **F)** Rhythm Method (Calendar Method)  **G)** Herbal Medications/Traditional (new option)  **H)** Other **(specify)**  __________________________ | 1 2  1 2  1 2  1 2  1 2  1 2  1 2 | Q77ATRT Q77ATRT  Q77BTRT Q77BTRT  Q77CTRT Q77CTRT  Q77DTRT Q77DTRT  Q77ETRT Q77ETRT  Q77FTRT Q77FTRT  QN77GTRT  Q77GTRT Q77GTRT  Q77GTRTS -------- |
| 66 | If you needed contraception, where would you get it?  **(Mark all that apply)** | **A)** Don’t know  **B)** Doctor’s office  **C)** Public health clinic  **D)** Youth clinic  **E)** Friends  **F)** Drug store, pharmacy, dispensary  **G)** Family planning clinic  **H)** Shop  I) Traditional Healer (new option)  **J)** Other (specify)  __________________________ | 1 2  1 2  1 2  1 2  1 2  1 2  1 2  1 2  1 2  1 2 | Q78ATRT Q78ATRT  Q78BTRT Q78BTRT  Q78CTRT Q78CTRT  Q78DTRT Q78DTRT  Q78ETRT Q78ETRT  Q78FTRT Q78FTRT  Q78GTRT Q78GTRT  Q78HTRT Q78HTRT  Q78ITRT  Q78JTRT Q78ITRT  Q78ITRTS ------- |

67. When did you last…

|  | Never/Don’t remember  **(1)** | In the past year  **(2)** | 1-2 years ago  **(3)** | Over 2 years ago  **(4)** |  |  |
| --- | --- | --- | --- | --- | --- | --- |
| a. Have a regular checkup (physical) |  |  |  |  | Q79ATRT | Q79ATRT |
| b. See a herbalist, traditional doctor or healer |  |  |  |  | Q79BTRT | Q79BTRT |
| c. Have your hearing checked |  |  |  |  | Q79CTRT | Q79CTRT |
| d. Have your eyes checked |  |  |  |  | Q79DTRT | Q79DTRT |
| e. See a dentist for your teeth |  |  |  |  | Q79ETRT | Q79ETRT |
| f. Get counseling or mental health  services (e.g. child guidance services for emotional problems) |  |  |  |  | Q79FTRT | Q79FTRT |

**MALARIA**

| 68 | How does someone get malaria  **(Check all that apply: 1=yes, 2=no)** | **A)** I don’t know  **B)** Bite from infected mosquitoes  **C)** Standing water  **D)** Long grass/dirty environment  **E)** From someone with malaria infection  **F)** Others (specify)  _________________________ | 1 2  1 2  1 2  1 2  1 2  1 2 | Q80ATRT Q80ATRT  Q80BTRT Q80BTRT  Q80CTRT Q80CTRT  Q80DTRT Q80DTRT  Q80ETRT Q80ETRT  Q80FTRT Q80FTRT  Q80FTRTS ------ |
| --- | --- | --- | --- | --- |
| 69 | Do you know of ways in which malaria can be prevented?  **(Check all that apply)** | **A)** Don’t know any  **B)**Use of mosquito/impregnated bednets  **C)**Mosquito repellents (including sprays, barks and creams)  **D)**Household spraying (from private company or municipal council)  **E)**Malaria prophylaxis  **F)**Intermittent presumptive treatment (IPT)  **G)**Removal of mosquito breeding sites  **H)**Others  _________________ | 1 2  1 2  1 2  1 2  1 2  1 2  1 2  1 2 | Q81ATRT Q81ATRT  Q81BTRT Q81BTRT  Q81CTRT Q81CTRT    Q81DTRT Q81DTRT  Q81ETRT Q81ETRT  Q81FTRT Q81FTRT  Q81GTRT Q81GTRT  Q81HTRT Q81HTRT  Q81HTRTS ------- |
| 70 | Which malaria preventative measures are usually practiced at your home?  **(Check all that apply)** | **A)** None  **B)** Use of mosquito/impregnated bed nets  **C)** Mosquito repellents (including sprays, barks and creams)  **D)** Household spraying (from private company or municipal council)  **E)** Malaria prophylaxis  **F)** Intermittent presumptive treatment (IPT)  **G)** Removal of mosquito breeding sites  **H)**Other  ________________________ | 1 2  1 2  1 2  1 2  1 2  1 2  1 2  1 2 | Q82ATRT Q82ATRT  Q82BTRT Q82BTRT  Q82CTRT Q82CTRT  Q82DTRT Q82DTRT  Q82ETRT Q82ETRT  Q82FTRT Q82FTRT  Q82GTRT Q82GTRT  Q82HTRT Q82HTRT  Q82HTRTS ------- |

HIV/AIDS

| 71 | Have you heard of an illness called AIDS | Yes  No  Refused | | | | | 01  02  99 | Q40SXL Q40SXL | |  |
| --- | --- | --- | --- | --- | --- | --- | --- | --- | --- | --- |
| 72 | How is AIDS spread?  **(Mark all that apply)** | | **A)** Sexual contact between two people, if one is infected  **B)** Touching, kissing, shaking hands (non-sexual contact)  **C)** Blood Transfusion of infected blood  **D)** Mother-to-Child Transmission, if mother is infected  **E)** Sharing of infected syringes, razors  **F)** Other (specify) ___________________________ | | 1 2  1 2  1 2  1 2  1 2  1 2 | | | Q41ASXL Q41ASXL  Q41BSXL Q41BSXL  Q41CSXL Q41CSXL  Q41DSXL Q41DSXL  Q41ESXL Q41ESXL  Q41FSXL Q41FSXL  Q41FSXLS ----- | |  |
| 73 | Is it possible for a healthy looking person to have AIDS? | Yes  No  Don’t know  Refused | | | | | 01  02  98  99 | Q42SXL Q42SXL | |  |
| 74 | Do you know anyone who is infected with HIV? | Yes  No  Don’t know  Refused | | | | | 01  02  98  99 | Q43SXL Q43SXL | |  |
|  |  |  | | | | |  |  | |  |
| 75 | Do you have any reason to believe that you are infected with HIV? | Yes, reason:  _______________________________  No  Don’t know  Refused | | | | | 01  02  98  99 | Q44SXL Q44SXL  Q44SXLY Q44SXLY | |  |
| 76 | Have you ever talked to someone about this [concerning HIV/AIDS infection]?  If yes, who did you speak with?  **(Mark all that apply)** | | | **A)** I’ve never spoken to anyone about it  **B)** Refused  **C)** A parent  **D)** A relative who lives with me  **E)** A relative who does not live with me  **F)** A brother, sister or other teenager who lives with me  **G)** A boyfriend, girlfriend or other teenager who does NOT live with me  **H)** A teacher  **I)** A pastor, sheik, or religious leader  **J)** A doctor  **K)** Any other adult | | 1 2  1 2  1 2  1 2  1 2  1 2  1 2  1 2  1 2  1 2  1 2 | | | Q45ASXL Q45ASXL  Q45BSXL Q45BSXL  Q45CSXL Q45CSXL  Q45DSXL Q45DSXL  Q45ESXL Q45ESXL  Q45FSXL Q45FSXL  Q45GSXL Q45GSXL  Q45HSXL Q45HSXL  Q45ISXL Q45ISXL  Q45JSXL Q45JSXL  Q45KSXL Q45KSXL | |
| 77 | Do you know about sexually transmitted diseases, such as syphilis, gonorrhea, chlamydia and herpes? | Yes  No  Refused | | | | | 01  02  99 | Q46SXL Q46SXL | |  |
| 78 | **(If sexually active)** Have you ever had such a problem? | Yes  No  Refused | | | | | 01  02  99 | Q47SXL Q47SXL | |  |

| **If a girl, go to question 81°--If a boy, go to question 82** | | | | |
| --- | --- | --- | --- | --- |
| **81a**  **93a** | (For Girls) Have you heard about female circumcision? | Yes (go to 81b)  No (skip to question 83)  Refused | 01  02  99 | Q48SXL Q48SXL |
| **93b** | Have you been circumcised? | Yes  No  Refused | 01  02  99 | Q50SXL Q50SXL |
| **93c** | If yes, at what age? (new question) | Give the age | -- | QN93CSXL |
| **94a** | (For boys)  Have you heard of circumcision? (new question) | Yes (go to 82b)  No (skip to 83)  Refused | 01  02  99 | QN94ASXL |
| **94b**  **94c** | If yes, have you been circumcised? (new question)  At what age? (new question) | Yes  No  Age_____ | 01  02 | QN94BSXL  QN94CSXL |
| **79** | Should a teacher with HIV be allowed to teach? (new question) | Yes  No  Don’t Know  Refused | 01  02  98  99 | QN79SXL |
| **80** | Should efforts be made to keep AIDS orphans in the community rather than placing them in institutions? (new question) | Institutions  Community  Don’t Know  Refused | 01  02  98  99 | QN80SXL |
| **81** | Should a parent who is HIV+ tell his/her child? (new question) | Yes  No  Don’t Know  Refused | 01  02  98  99 | QN81SXL |
| **82** | Would you eat at the home where someone in the family has AIDS? (new question) | Yes  No  Don’t Know  Refused | 01  02  98  99 | QN82SXL |
| **83** | Can the virus that causes AIDS be spread from mother to child during pregnancy? (new question) | Yes  No  Don’t Know  Refused | 01  02  98  99 | QN83SXL |
| **84** | What does the HIV/AIDS test look for in the blood to determine if a person is HIV positive? (new question) | Virus  Antibody  Red Blood Cells  Germs  Don’t Know  Refused | 01  02  03  04  98  99 | QN84SXL |
| **89** | Do the HIV/AIDS drugs completely destroy the virus in the blood? (new question) | Yes  No  Don’t Know  Refused | 01  02  98  99 | QN85SXL |
| **90** | Can HIV/AIDS drugs cure a person who has HIV/AIDS? (new question) | Yes, they cure  No, they don’t cure  Don’t Know  Refused | 01  02  98  99 | QN86SXL |
|  |  |  |  |  |
|  |  |  |  |  |
|  |  |  |  |  |

PUBERTAL DEVELOPMENT AND NUTRITION

| 91  87 | Would you say your growth in height……?  **(Mark one box)** | Has not yet begun to spurt *(“spurt” means more growth than usual)*  Has barely started  Is definitely underway  Seems completed | | | 01  02  03  04 | Q83GRD Q83GRD |
| --- | --- | --- | --- | --- | --- | --- |
| 92  88 | And how about the growth of body hair? (Body hair means underarm or pubic  hair.) Would you say that your body hair has? **(Mark one box)** | Not yet started growing  Has barely started growing  Is definitely underway  Seems completed | | | 01  02  03  04 | Q84GRD Q84GRD |
| 93  89 | Have you noticed any skin changes, especially pimples? **(Mark one box)** | Not yet started showing changes  Have barely started showing any changes yet  Skin changes are definitely underway  Skin changes seem completed | | | 01  02  03  04 | Q85GRD Q85GRD |
|  | **For GIRLS Only** |  | | |  |  |
| 94a  90a | Have your breasts begun to grow?  (**Mark one box)** | Not yet started growing  Have barely started changing  Breast growth is definitely underway  Breast growth seems completed | | | 01  02  03  04 | Q86AGRD Q86AGRD |
| 94b  90b | Do you think your development is any earlier or later than most other girls your age?  **(Mark one box)** | Much earlier  Somewhat earlier  About the same time  Somewhat later  Much later  Doesn’t apply to me | | | 01  02  03  04  05  06 | Q87AGRD Q87AGRD |
| 94c  90c | Have you begun to menstruate or get you period? | Yes  No **(skip to question # 90)** | | | 01  02 | Q88AGRD Q88AGRD |
| 94d  90d | How old were you when you first menstruated or got your period? | | | Years | _ _ | Q89AGRD Q89AGRD |
|  | **For BOYS Only** |  | | |  |  |
| 95a  91a | Have you begun to grown hair on your face?  **(Mark one box)** | Not yet started growing hair  Has barely started growing hair  Facial hair growth is definitely underway  Facial hair growth seems completed | | | 01  02  03  04 | Q86BGRD Q86BGRD |
| 95b  91B | Do you think your development is any earlier or later than most other boys your age? **(Mark one box)** | Much earlier  Somewhat earlier  About the same  Somewhat later  Much later | | | 01  02  03  04  05 | Q87BGRD Q87BGRD |
| 95c  91c | Have you noticed a deepening of your voice?  **(Mark one box)** | Not yet started changing  Has barely started showing any changes  Voice change is definitely underway  Voice change seems completed | | | 01  02  03  04 | Q88BGRD Q88BGRD |
|  | **ALL RESPONDENTS** |  | | |  |  |
| 95d  92a | Have anybody told you what happens when children reach puberty? (new question) | | Yes  No | | 01  02 | QN92AGRD |
| 95e  92b | If yes, who told you what happens? (new question) | | Mother  Father  Grandmother  Grandfather  Aunt  Sister  Neighbor  Teacher  Doctor  Other | | 01  02  03  04  05  06  07  08  09  10 | QN92BGRD  QN92BGRS |
|  |  | |  | |  |  |
|  |  | |  | |  |  |
| 96c  95 | Do you think your weight it…..? | About right  Not sure  I need to gain weight  I need to loose weight | | | 01  02  03  04 | Q90GRD Q90GRD |
| 97  96 | When you look at yourself in the mirror, in general you feel……? | Not happy with the way your body looks  Just ok with the way your body looks  Happy with the way your body looks | | | 01  02  03 | Q91GRD Q91GRD |
| 98  97 | How many meals do you eat a day? | 0-1  2  3  4 or more | | | 01  02  03  04 | Q92GRD Q92GRD |

**SELF-EFFICACY**

**(For each question, read *“I have learned…”* before each statement. The child should indicate the degree to which she/he has learned that skill, NO, SOME, or YES. Interviewer, please mark X in the appropriate box.)**

| **99 Academics**:  98:  *I have learned…* |  | | | NO  **(1)** | | | SOME  **(2)** | | | YES  **(3)** | | | N/A  **(4)** | | |  | |  | |
| --- | --- | --- | --- | --- | --- | --- | --- | --- | --- | --- | --- | --- | --- | --- | --- | --- | --- | --- | --- |
| a) How to do my best on tests and exams. | |  | |  | | |  | | |  | | |  | | | Q93ASLE | | Q93ASLE | |
| b) How hard work helps me do well in math. | |  | |  | | |  | | |  | | |  | | | Q93BSLE | | Q93BSLE | |
| c) How to start and finish my homework. | |  | |  | | |  | | |  | | |  | | | Q93CSLE | | Q93CSLE | |
| d) That trying my best helps me do better in language studies. | |  | |  | | |  | | |  | | |  | | | Q93DSLE | | Q93DSLE | |
| **100 Deliberation:**  **99**  *I have learned…….* | | | NO  **(1)** | | SOME  **(2)** | | | YES  **(3)** | | | N/A  **(4)** | | |  | | |  | |  |
| a) How to express my opinions to other children in my age group. | | |  | |  | | |  | | |  | | | Q94ASLE | | | Q94ASLE | |  |
| b) How to make my ideas understood even when other children disagree with me. | | |  | |  | | |  | | |  | | | Q94BSLE | | | Q94BSLE | |  |
| c) That other children and adults will value me even if they disagree with my opinions. | | |  | |  | | |  | | |  | | | Q94CSLE | | | Q94CSLE | |  |
| d) How to use talking and reasoning to solve difficult problems I have with other children | | |  | |  | | |  | | |  | | | Q94DSLE | | | Q94DSLE | |  |
| e) How to express my opinion with adults (new question) | | |  | |  | | |  | | |  | | | Q94ESLE | | |  | |  |
| **101 Peer Relationships:**  **100**  *I have learned…….* | | | NO  **(1)** | | | SOME  **(2)** | | | YES  **(3)** | | | N/A  **(4)** | | |  | | |  | |
| a) How to be a good friend. | | |  | | |  | | |  | | |  | | | Q95ASLE | | | Q95ASLE | |
| b) How to understand my friends and classmates. | | |  | | |  | | |  | | |  | | | Q95BSLE | | | Q95BSLE | |
| c) How to keep friends even when they pressure me to do things I refuse to do. | | |  | | |  | | |  | | |  | | | Q95CSLE | | | Q95CSLE | |
| d) How to say NO to my friends when they want me to do things I don’t want to do. | | |  | | |  | | |  | | |  | | | Q95DSLE | | | Q95DSLE | |
| **102 Knowledge of Self and Others:**  **101**  *I have learned…….* | | | NO  **(1)** | | SOME  **(2)** | | | | YES  **(3)** | | | N/A  **(4)** | | |  | | |  | |
| a) How to manage my feelings. | | |  | |  | | | |  | | |  | | | Q96ASLE | | | Q96ASLE | |
| b) How to express those thoughts and feelings that are important to me. | | |  | |  | | | |  | | |  | | | Q96BSLE | | | Q96BSLE | |
| c) How to handle my problems. | | |  | |  | | | |  | | |  | | | Q96CSLE | | | Q96CSLE | |
| d) About my strengths and talents. | | |  | |  | | | |  | | |  | | | Q96DSLE | | | Q96DSLE | |
| e) How to understand and accept myself. | | |  | |  | | | |  | | |  | | | Q96ESLE | | | Q96ESLE | |
| **103 Health:**  **102**  *I have learned…….* | | | NO  **(1)** | | SOME  **(2)** | | | | YES  **(3)** | | | N/A  **(4)** | | |  | | |  | |
| a) How to protect my health. | | |  | |  | | | |  | | |  | | | Q97ASLE | | | Q97ASLE | |
| b) About the right kinds of food to eat to stay healthy. | | |  | |  | | | |  | | |  | | | Q97BSLE | | | Q97BSLE | |
| c) How to say NO to people who try to get me to use alcohol. | | |  | |  | | | |  | | |  | | | Q97CSLE | | | Q97CSLE | |
| d) How to say NO to sex when I don’t want to have sex. | | |  | |  | | | |  | | |  | | | Q97DSLE | | | Q97DSLE | |
| e) How to ask adults for advice on sexually transmitted diseases (STDs). | | |  | |  | | | |  | | |  | | | Q97ESLE | | | Q97ESLE | |
| **104 Relationship between Child and Adult:**  **103**  *In the last 12 months I have learned……. (new section)* | | | NO  **(1)** | | SOME  **(2)** | | | | YES  **(3)** | | | N/A  **(4)** | | |  | | |  | |
| a) How to make adults listen to me | | |  | |  | | | |  | | |  | | | QN103ASL | | |  | |
| b) How to ask for assistance from adults about how to solve any problem that I may have | | |  | |  | | | |  | | |  | | | QN103BSL | | |  | |
| c) How to talk with others on any issue or matter that disturbs me or troubles me | | |  | |  | | | |  | | |  | | | QN103CSL | | |  | |
| d) How to make others cooperate with me in doing activities that I do | | |  | |  | | | |  | | |  | | | QN103DSL | | |  | |

| **105** | Now, I would like to ask you several questions in order to know whether you think you have changed during the past 12 months. We will go through some areas and if you think there have been changes in your ability to express your opinions and to be understood, please tell me. (new section) |  |  |  |
| --- | --- | --- | --- | --- |
| **105a**  **104a** | In the past 12 months, have you changed in your ability to talk with your parents about your worries? (Read the answers)  Comment: | Talk with them more  Talk with them less  No change | 01  02  03 | QN104ACH  QN104ACS |
| **105b**  **104b** | During the past 12 months, has your ability to express yourself and be understood made physical punishment against you be less or more? (Read the answers)  Comment: | Punished more  Punished less  No change | 01  02  03 | QN104BCH  QN104BCS |
| **105c**  **104c** | In the past 12 months, have you changed in your ability to express yourself and be understood by your peers? (Read the answers)  Comment: | Talk with them more  Talk with them less  No change | 01  02  03 | QN104CCH  QN104CCS |
| **105d**  **104d** | In the past 12 months, have you changed in your ability to express yourself and be understood by teachers? (Read the answers)  Comment:’ | Talk with them more  Talk with them less  No change | 01  02  03 | QN104DCH  QN104DCS |
| **105e**  **104e** | In the past 12 months, have you changed in your ability to express yourself and be understood by other adults? | Talk with them more  Talk with them less  No change | 01  02  03 | QN104ECH  QN104ECS |
|  |  |  |  |  |
| **104f** | In the past 12 months, have you changed in your ability to express yourself and be understood by your siblings? | Talk with them more  Talk with them less  No change | 01  02  03 | QN104FCH  QN104FCS |

***“Thank you for your cooperation.”***

**ANTHROPOMETRICS**

| 1^st^ Assessment | | | | |
| --- | --- | --- | --- | --- |
| 102  105 | HEIGHT |  | **__ __ __ . __ cm** | QN105HGT |
| 103  106 | WEIGHT |  | **__ __ __ . __ kg** | QN106WGT |
| 104  107 | MUAC |  | **__ __ __ mm** | QN107MUC |
| 2^nd^ Assessment | | | | |
| 102  108 | HEIGHT |  | **__ __ __ . __ cm** | QN108HG2 |
| 103  109 | WEIGHT |  | **__ __ __ . __ kg** | QN109WG2 |
| 104  110 | MUAC |  | **__ __ __ mm** | QN110MU2 |
| 3^rd^ Assessment—By Supervisor | | | | |
| 102  111 | HEIGHT |  | **__ __ __ . __ cm** | QN111HG3 |
| 103  112 | WEIGHT |  | **__ __ __ . __ kg** | QN112WG3 |
| 104  113 | MUAC |  | **__ __ __ mm** | QN113MU3 |

**ASSESSMENT DEBRIEFING**

| 114 | Was the interview completed? | Yes  No | 1  2 | Q105BRPT | Q105BRPT |
| --- | --- | --- | --- | --- | --- |
| 115 | If not, was this because the child: | Could not interview  Refused  Other:_____**___________** | 1  2  3 | Q106BRPT  Q106BRPO | Q106BRPT  Q106BRPTOT |
| 107  116 | Did the child understand most questions? | Yes  No | 1  2 | Q107BRPT | Q107BRPT |
| 108  117 | How cooperative was the child? | Very  Somewhat  Not cooperative | 1  2  3 | Q108BRPT | Q108BRPT |
| 109  118 | Do you think the child answered all sections honestly? | Yes  Some  No | 1  2  3 | Q109BRPT | Q109BRPT |
| 110  119 | If no, which sections made you think this? | **(Write number of section(s) here)** |  | Q110BRPT | Q110BRPT |
| 111  120 | Were there any problems during the interview because of the presence of others? | Yes  No | 1  2 | Q111BRPT | Q111BRPT |
| 121 | Did any thing happen during interview that made you think that a child participated in CHASE project? (new question)  If yes (explain) | Yes  No | 1  2 | Q121BRPT  Q121BRPO |  |
|  | General comments |  |  | Q121X |  |
