# Supplementary material for: Rural-urban disparities in the nutritional status of younger adolescents in Tanzania
Source: PLoS One. 2021 Dec 20;16(12):e0261480. doi: 10.1371/journal.pone.0261480 (PMC8687541; doi:10.1371/journal.pone.0261480)
Supplement: S3 File — (DOC) [file pone.0261480.s003.doc]

**UNICEF BASELINE SURVEY 2003-2004**

LATEST VERSION OF ADOLESCENT MODULE

10/2005

**Round 1**

**(December 2003 – February 2004)**

**ADOLESCENT MODULE**

**Questionnaire for Adolescent Respondent - 2**

| 1. **Identification**   1.1 Household number **----------------------------------------------------------------------------** 1.2 Name of Household head -------------------------------------------------------------------- 1.3 Adolescent’s Member ID --------------------------------------------------------------------  1.4 Name of Hamlet -----------------------------------------------------------------------------  1.5 Name of village/cluster ----------------------------------------------------------------------  1.6 Name of Ward ---------------------------------------------------------------------------------  1.7 Name of District -----------------------------KILOSA---------------------------------------  1.8 Name of Interviewer/Enumerator------------------------------------------------------------  1.9 Name of Supervisor -------------------------------------------------------------------------- | **Comments** |
| --- | --- |

Date of Interview

| 1^st^ Time | | | 2^nd^ Time | | | 3^rd^ Time | | |
| --- | --- | --- | --- | --- | --- | --- | --- | --- |
| Day | Month | Year | Day | Month | Year | Day | Month | Year |
|  |  |  |  |  |  |  |  |  |

Start Time: __ __ __:__ __ __ Finish Time: __ __ __:__ __ __

**2a. Anthropometry**

Note: Complete columns for adolescents (10-19 years):

Was adolescent measured? Yes….1

No…..2 *(if no, why) _______________________________________*

| Member ID | Name | Height (cm)  **#1** | Height (cm)  **#2** | Weight (kg)  **#1** | Weight (kg)  **#2** | MUAC  (cm)  **#1** | MUAC (cm)  **#2** | Pregnant?  Yes….1  No…..2  DK….3 | If yes, duration of pregnancy  (months) | Lactating?  Yes….1 No…..2 | Duration of lactation  (month) |
| --- | --- | --- | --- | --- | --- | --- | --- | --- | --- | --- | --- |
| a | b | c | d | e | f | g | h | i | j | k | l |
|  |  |  |  |  |  |  |  |  |  |  |  |
|  |  |  |  |  |  |  |  |  |  |  |  |

**2b. Previous Health Status**

(1) (2)

Have you lived in this household for the entire past 12 months? ____Yes→2c ____No

If not, how would you rate your health status in the time immediately preceding your arrival into the current household? (1 is excellent health and 5 is very poor health status): *Note: Circle response.*

Very Don’t

Excellent Poor Know 1 2 3 4 5 6

2c. Demographics:

| Member  ID | Name | Sex  M…1  F….2 | Date of Birth* | | Marital Status  (code) | Currently enrolled in school?  Yes…1→ h  No.…2→i | *C*urrent grade or COBET level→k    Dk…3 | Last grade level→ j  DK…3 | If not attending school,  primary reason why?  (code) | ***Girls Only:***  Age of menarche  Not yet…….2  Don’t remember..999  (Years) | ***Boys Only:***  Age of first voice change  Not yet changed…2  Don’t remember..999  (Years) |
| --- | --- | --- | --- | --- | --- | --- | --- | --- | --- | --- | --- |
|  |  |  | Month | Year |  |  |  |  |  |  |  |
| a | b | c | d | e | f | G | h | i | j | k | l |
|  |  |  |  |  |  |  |  |  |  |  |  |

* *If person does not know Date of Birth, put code: 999*

**3. Labor Participation:**

Now I am going to ask you a few questions about work

| Have you worked in the past month? This includes work in the formal and informal sectors.  *If Yes,* for pay?  Yes, for Pay (Cash or Kind)...1  Yes, Unpaid…………………2  No….……...3 → Next section (Health Status) | What type of labor or work did you do?    (code) | How many hours per day did you work? *If more than one job, include all hours at all jobs.*  (No. of hours) | How many days did you work in the past month?  (No. of days) | How many months did you work in the past year?  (No. of months) |
| --- | --- | --- | --- | --- |
| a | b | c | d | e |
|  |  |  |  |  |

| **Marital Status code:**  Never married………………………..1  Married ……………………2  Separated ……………………3  Divorced ……………………4  Widowed ……………………5 | **Reason not attending school code:**  Pregnancy……………………….…1  Lack of uniform, school supplies.…2  School full/no place……………..…3  Death of parent/guardian………..…4  Early marriage…………………..…5  Adult in household sick/disabled….6  Ill/disability (self)..……………..…7  Corporal punishment………………8  Need to work ……………….…9  School too far……………….….…10  Did not pass……………………….11  Other (specify)…………….………12  Don’t know…………………….…13 | **Type of labor code:**  Farming, forestry, fishing………….……..1  Herder, pastoralist………………………...2  Non-agri day labor………….…………….3  Professional/Clerical……………………...4  Service (tailor, hairdresser, mechanic)……5  Domestic servant.…………………………6  Vendor (vegetable, products)……………..7  Beggar …………………………8  Mining…..………………………..……….9  Commercial sex worker………………….10  Work in a bar…………………………….11  Does not work……………………………12  Other……………………………………..13 |
| --- | --- | --- |

**4. Dietary Recall: Last 24 Hours**

***(Gather information on last 24 hours individual food consumption or the last “typical”eating day)***

**Cooking method (code):**

Raw..……….0 Stew..………3

Boil…………1 Kuoka………4

Fry…….……2 Roast………..5

| **Recall Date:** | Day | Month | Year | **Respondent ID:** |  |
| --- | --- | --- | --- | --- | --- |
|  |  |  |

| Time that respondent ate or drank | Meal (menu) | Meal (menu)  (code) | Quantity | | | Ingredient | Ingredient | | Source of Food:  Left over from previous day …1  Food cooked in the HH …2  Purchased …3  Food received from others …4  Invitation …5  Food taken at employers place...6 |
| --- | --- | --- | --- | --- | --- | --- | --- | --- | --- |
|  |  |  | Amount | Measurement | Type of measurement  (code) |  | Cooking method  (code) | Ingredient  (code) |  |
| a | b | c | d | e | f | g | h | i | j |
|  |  |  |  |  |  |  |  |  |  |
|  |  |  |  |  |  |  |  |  |  |
|  |  |  |  |  |  |  |  |  |  |
|  |  |  |  |  |  |  |  |  |  |
|  |  |  |  |  |  |  |  |  |  |
|  |  |  |  |  |  |  |  |  |  |
|  |  |  |  |  |  |  |  |  |  |
|  |  |  |  |  |  |  |  |  |  |
|  |  |  |  |  |  |  |  |  |  |
|  |  |  |  |  |  |  |  |  |  |
|  |  |  |  |  |  |  |  |  |  |
|  |  |  |  |  |  |  |  |  |  |
|  |  |  |  |  |  |  |  |  |  |
|  |  |  |  |  |  |  |  |  |  |
|  |  |  |  |  |  |  |  |  |  |

**4. Dietary Recall: Last 24 Hours**

***(Gather information on last 24 hours individual food consumption or the last “typical”eating day)***

**Cooking method (code):**

Raw..……….0 Stew..………3

Boil…………1 Kuoka………4

Fry…….……2 Roast………..5

| **Recall Date:** | Day | Month | Year | **Respondent ID:** |  |
| --- | --- | --- | --- | --- | --- |
|  |  |  |

| Time that respondent ate or drank | Meal (menu) | Meal (menu)  (code) | Quantity | | | Ingredient | Ingredient | | Source of Food:  Left over from previous day …1  Food cooked in the HH …2  Purchased …3  Food received from others …4  Invitation …5  Food taken at employers place...6 |
| --- | --- | --- | --- | --- | --- | --- | --- | --- | --- |
|  |  |  | Amount | Measurement | Type of measurement  (code) |  | Cooking method  (code) | Ingredient  (code) |  |
| a | b | c | d | e | f | g | h | i | j |
|  |  |  |  |  |  |  |  |  |  |
|  |  |  |  |  |  |  |  |  |  |
|  |  |  |  |  |  |  |  |  |  |
|  |  |  |  |  |  |  |  |  |  |
|  |  |  |  |  |  |  |  |  |  |
|  |  |  |  |  |  |  |  |  |  |
|  |  |  |  |  |  |  |  |  |  |
|  |  |  |  |  |  |  |  |  |  |
|  |  |  |  |  |  |  |  |  |  |
|  |  |  |  |  |  |  |  |  |  |
|  |  |  |  |  |  |  |  |  |  |
|  |  |  |  |  |  |  |  |  |  |
|  |  |  |  |  |  |  |  |  |  |
|  |  |  |  |  |  |  |  |  |  |
|  |  |  |  |  |  |  |  |  |  |

**4. Dietary Recall: Last 24 Hours**

***(Gather information on last 24 hours individual food consumption or the last “typical”eating day)***

**Cooking method (code):**

Raw..……….0 Stew..………3

Boil…………1 Kuoka………4

Fry…….……2 Roast………..5

| **Recall Date:** | Day | Month | Year | **Respondent ID:** |  |
| --- | --- | --- | --- | --- | --- |
|  |  |  |

| Time that respondent ate or drank | Meal (menu) | Meal (menu)  (code) | Quantity | | | Ingredient | Ingredient | | Source of Food:  Left over from previous day …1  Food cooked in the HH …2  Purchased …3  Food received from others …4  Invitation …5  Food taken at employers place...6 |
| --- | --- | --- | --- | --- | --- | --- | --- | --- | --- |
|  |  |  | Amount | Measurement | Type of measurement  (code) |  | Cooking method  (code) | Ingredient  (code) |  |
| a | b | c | d | e | f | g | h | i | j |
|  |  |  |  |  |  |  |  |  |  |
|  |  |  |  |  |  |  |  |  |  |
|  |  |  |  |  |  |  |  |  |  |
|  |  |  |  |  |  |  |  |  |  |
|  |  |  |  |  |  |  |  |  |  |
|  |  |  |  |  |  |  |  |  |  |
|  |  |  |  |  |  |  |  |  |  |
|  |  |  |  |  |  |  |  |  |  |
|  |  |  |  |  |  |  |  |  |  |
|  |  |  |  |  |  |  |  |  |  |
|  |  |  |  |  |  |  |  |  |  |
|  |  |  |  |  |  |  |  |  |  |
|  |  |  |  |  |  |  |  |  |  |
|  |  |  |  |  |  |  |  |  |  |
|  |  |  |  |  |  |  |  |  |  |

*Source: Tufts University, FANTA Project*

5a. Health Status

People in our community get sick from time to time. The following questions are about illness **you** might have experienced:

| Persistent diarrhoea now or two or more episodes of acute diarrhoea in past 3 months  Definition: *3 + watery stools in a 24 hour period*  Yes…..1  No…...2  DK…..3 | Two or more episodes of cough, rapid breathing, wheezing, and fever in last 2 months?  Yes…1 → c  No….2 →d  DK…3 → d | Did a doctor give you antibiotics for this illness?  Yes…..1  No…...2  DK……3  Did not go…4 | Episodes  of thrush or fungal infection of the mouth in the past 3 months?*  Yes…..1  No…..2  DK….3 | Swelling of glands in the mouth and throat in the past 3 months?  Yes….1  No…..2  DK….3 | Did you lose a lot of weight in the past 12 months?  Yes....1→ g  No…..2→h  DK….3→h | If you lost a lot of weight, have you been able to gain back weight in the past 12 months?  Yes....1  No…..2  DK….3 | Episodes of ear discharge now or at any time in the past 12 months?  Yes…..1  No…...2  DK…..3 | Did person have swelling, lumps, tenderness and/or pain in two or more of these sites: neck, groin, and armpit?  Yes…..1  No…..2  DK…..3 |
| --- | --- | --- | --- | --- | --- | --- | --- | --- |
| a | b | c | d | e | f | g | h | i |
|  |  |  |  |  |  |  |  |  |

Source: WHO

* Symptoms of thrush or fungal infection of the mouth include discomfort and burning of the mouth and throat; an altered sense of taste (often described as "bad"); creamy white or yellowish spots on or around mouth.

**5b. Health Status: Disability, HIV/AIDS, Malaria**

In the past year, have you been sick/disabled for more than 1 month? Yes….1

No…..2 *(if no, go directly to column e in the box below)*

| Member Id | For how many days have you been sick/disabled?  (No. of Days) | What type of sickness or disability do/did you have?  If HIV/AIDS is given as a sickness → d  Otherwise→e | If HIV/AIDS, how did you know that you had HIV/AIDS?  (code) | Have you had tuberculosis in the past year?  Yes….1  No…..2  DK…..3 | Did you have malaria in last 3 months?  Yes...1→ g  No….2 → h  DK…3 → h | How did you know you had malaria?  (code) | Did you receive medical treatment any illness that you had?  Yes….1→ j No….2→ i  DK….3→ i  N/A…4→ i | If no, was this due to lack of money?  Yes…1  No….2  DK…3  N/A….4 | Before getting ill, were you the primary income earner?  Yes….1  No…..2  No response…..3  N/A....4 | Did you earn less income than usual after becoming sick?  Yes….1  No…..2  No response…..3  N/A....4 |
| --- | --- | --- | --- | --- | --- | --- | --- | --- | --- | --- |
| a | b | c | d | e | f | g | h | i | j | k |
|  |  |  |  |  |  |  |  |  |  |  |

| **How did person know it was HIV/AIDS code :**  Doctor/clinic/VCT diagnosed with blood test...….1  Doctor/clinic/VCT diagnosed w/o blood test…….2  Traditional doctor………………………………...3  Someone other than a doctor assumed……….…..4  I assumed based on symptoms……………………5  Other …………………..6 | **How did person know it was Malaria code :** Doctor/clinic/VCT diagnosed with blood test...….1  Doctor/clinic/VCT diagnosed w/o blood test…….2  Traditional doctor………………………………...3  Someone other than a doctor assumed……….…..4  I assumed based on symptoms……………………5 Other …………………..6 |
| --- | --- |

**5c. This next question will be about whether you are concerned that anyone in your household has HIV/AIDS. I would like to remind you that you do not have to respond**

**to this question, and that this information will be kept confidential if you do respond.**

#### Are you worried that anyone in your household may have HIV/AIDS now? ❑ Yes ❑ No ❑ Don’t Know ❑ No Response

(1) (2) (3) (4)

**6. Hygiene and Sanitation**

| What is your main source of drinking water?  (code) | How often do you drink boiled or bottled water?  (code) | What kind of toilet facility do you use?  (code) | People find it necessary to wash their hands before or after certain activities.  When do you wash your hands? | | | | |
| --- | --- | --- | --- | --- | --- | --- | --- |
|  |  |  | After defecation  Yes….1  No…..2  N/A….3 | Before food preparation  Yes….1  No…..2  N/A….3 | Before eating  Yes….1  No…..2  N/A….3 | After cleaning babies bottoms  Yes….1  No…..2  N/A….3 | Before feeding children  Yes….1  No…..2  N/A….3 |
| a | b | c | d | e | f | g | h |
|  |  |  |  |  |  |  |  |

| **Main source of drinking water code :**  Supply/piped water……………………………...1  Tubewell/borehole with pump…………………..2  Well (unprotected)…………………………….…3  Rainwater collection……………………………..4  Pond, river, stream……………………………….5  Other……………………………………………..6  No response………………………………………7 | **How often drink boiled/bottled water code:**  Always 1  Sometimes 2  Only when sick 3  Never 4  Other 5 | **Toilet facility used code:**  Flush 1  Improved Pit Latrine (VIP) 2  Traditional Pit Latrine 3  Open pit 4  Bucket 5  No facilities or bush or field 6  Other (specify) 7 |
| --- | --- | --- |

1. Orphanhood and Property-grabbing

*If both parents are alive, please skip to next section (7a) on Physical Activity.*

| Is your mother alive? | | Is your father alive? | | *If one or both parents died:*  Since your parent(s) died, have you had any property stolen from you?  Yes…1  No….2  DK…3 | What type of property was taken?  (code) | Who took your property?  (code) | How many of your siblings (less than 19 years) **do not** live in this household?  (number) |
| --- | --- | --- | --- | --- | --- | --- | --- |
| Yes…1  No….2→ c  DK…3→c | *If No:* Year of Death | Yes…1 No….2→e  DK…3→e | *If No:*  Year of Death |
| a | b | c | d | e | f | g | h |
|  |  |  |  |  |  |  |  |

| **Type of property taken code:**  Land……………………………………………………….………….1  Housing…………………………………………………………….…2  Money………………….……………………………………………..3  Animals……………………………………………………………….4  Material property (furniture, kitchen utensils, etc.)…………………..5  Other (Specify)….…………………………………………………….6 | **Who took your property code:**  Relatives……………………………..1  Guardian……………………………...2  Neighbors…………………………….3  Community…………………………..4  Other………………………………….5  Don’t know…………………………..6 |
| --- | --- |

**8. Physical Activity Recall**

I am going to ask you about the time you spent being physically active in the last 7 days. Please answer each question even if you do not consider yourself to be an active person. Think about the activities you do at work, as part of your house and yard work, to get from place to place, and in your spare time for recreation, exercise or sport.

**8a. Vigorous Activities:**

Now, think about all the *vigorous* activities which take *hard physical effort* that you did in the last 7 days. Vigorous activities make you breathe much harder than normal and may include heavy lifting, digging, football, or fast bicycling. Think only about those physical activities that you did for at least 10 minutes at a time.

| During the **last 7 days**, on how many days did you do **vigorous** physical activities? | Did you do vigorous physical activity yesterday? | **Interviewer clarification**: Think only about those physical activities that you do for at least 10 minutes at a time. (*If respondent answers zero, refuses or does not know, skip to* **8b**) | | **Interviewer clarification**: Think only about those physical activities you do for at least 10 minutes at a time. |
| --- | --- | --- | --- | --- |
|  |  | How much time did you usually spend doing **vigorous** physical activities on one of those days? | How much time did you spend doing vigorous physical activities yesterday? | **Interviewer probe**: An average time for one of the days on which you do vigorous activity is being sought. *If the respondent can't answer because the pattern of time spent varies widely from day to day, ask*: "How much time in total would you spend **over the last 7 days** doing vigorous physical activities?” |
| a | b | c | d | e |
| ____ No. days/wk  ____ DK/Not Sure  (999) | ___ Yes (1)  ___ No (2) | __ __ Hours per day  __ __ __ Minutes per day  _______ DK/Not Sure (999) | __ __ Hours per day  __ __ __ Minutes per day  _______ DK/Not Sure (999) | __ __ Hours per week  __ __ __ Minutes per week  _______ DK/Not Sure (999) . |

**8b. Moderate Activities:**

Now think about activities which take *moderate physical effort* that you did in the last 7 days. Moderate physical activities make you breathe somewhat harder than normal and may include carrying light loads, bicycling at a regular pace, or volleyball. Do not include walking. Again, think about only those physical activities that you did for at least 10 minutes at a time.

| During the **last 7 days**, on how many days did you do **moderate** physical activities? | Did you do moderate physical activities yesterday? | **Interviewer clarification**: Think only about those physical activities that you do for at least 10 minutes at a time. (*If respondent answers zero, refuses or does not know, skip to Question* **8c***)* | | **Interviewer clarification**: Think only about those physical activities that you do for at least 10 minutes at a time. |
| --- | --- | --- | --- | --- |
|  |  | How much time did you usually spend doing **moderate** physical activities on one of those days? | How much time did you spend doing moderate physical activities yesterday? | **Interviewer probe**: An average time for one of the days on which you do moderate activity is being sought. *If the respondent can't answer because the pattern of time spent varies widely from day to day, or includes time spent in multiple jobs, ask*: “What is the total amount of time you spent over the **last 7 days** doing moderate physical activities?” |
| a | b | c | d | e |
| ____ No. days/wk  ____ DK/Not Sure  (999) | ___ Yes (1)  ___ No (2) | __ __ Hours per day  __ __ __ Minutes per day  _______ DK/Not Sure (999) | __ __ Hours per day  __ __ __ Minutes per day  _______ DK/Not Sure (999) | __ __ Hours per week  __ __ __ Minutes per week  _______ DK/Not Sure (999) . |

**8c. Light Activities:**

Now think about the time you spent walking in the last 7 days. This includes at work and at home, walking to and from work, traveling from place to place, and any other walking that you might do solely for recreation, sport, exercise, or leisure.

| During the **last 7 days**, on how many days did you **walk** for at least 10 minutes at a time? | Yesterday, did you **walk** for at least 10 minutes at a time? | **Interviewer clarification**: Think only about the walking that you do for at least 10 minutes at a time. (*If respondent answers zero, refuses or does not know, skip to Question* **8d***)* | | **Interviewer probe**: An average time for one of the days on which you walk is being sought. *If the respondent can't answer because the pattern of time spent varies widely from day to day, ask:* “What is the total amount of time you spent walking over **the last 7 days**?” |
| --- | --- | --- | --- | --- |
|  |  | How much time did you usually spend **walking** on one of those days? | How much time did you spend walking yesterday? |  |
| a | b | c | d | e |
| ____ No. days/wk  ____ DK/Not Sure  (999) | ___ Yes (1)  ___ No (2) | __ __ Hours per day  __ __ __ Minutes per day  _______ DK/Not Sure (999) | __ __ Hours per day  __ __ __ Minutes per day  _______ DK/Not Sure (999) | __ __ Hours per week  __ __ __ Minutes per week  _______ DK/Not Sure (999) |

**8d. Sedentary Activities:**

Now think about the time you spent sitting on week days during the last 7 days. Include time spent at work, at home, while doing course work, and during leisure time. This may include time spent sitting at a desk, visiting friends, reading or sitting or lying down to listen to radio or watch television.

| During the last 7 days, how much time did you usually spend ***sitting*** on a **week day**? | **Interviewer clarification**: Include time spent lying down (awake) as well as sitting |
| --- | --- |
|  | **Interviewer probe**: An average time per day spent sitting is being sought*. If the respondent can't answer because the pattern of time spent varies widely from day to day, ask*: “What is the total amount of time you spent *sitting* **yesterday**?” |
| a | b |
| __ __ Hours per day  __ __ __ Minutes per day  _______ DK/Not Sure (999) | __ __ Hours yesterday  __ __ __ Minutes yesterday  _______ DK/Not Sure (999) . |

*Source: International Physical Activity Questionnaire, WHO*

| **9. Food Frequency** | | Every Day  (1) | 3 - 6 times/wk  (2) | 1-2 times/wk  (3) | Less than once/wk  (4) | Never  (5) |
| --- | --- | --- | --- | --- | --- | --- |
| a. | In the last 7 days, how often did you eat less food than usual due to scarcity of food? | ❑ | ❑ | ❑ | ❑ | ❑ |
| b. | In the last 7 days, how often did you skip entire meals because there was no food? (not including days that you were sick) | ❑ | ❑ | ❑ | ❑ | ❑ |
| c. | In the last 7 days, how many times did you eat meat or fish? | ❑ | ❑ | ❑ | ❑ | ❑ |
| d. | In the last 7 days, how many times did you eat green leafy vegetables? | ❑ | ❑ | ❑ | ❑ | ❑ |
| e. | In the last 7 days, how many times did you eat pumpkin, carrots, mango, or papaya? | ❑ | ❑ | ❑ | ❑ | ❑ |

*Source: Tufts University FANTA Project*

| **10. RISK/PROTECTIVE FACTORS MODULE** | Always  (1) | Often  (2) | Some-times  (3) | Rarely  (4) | Never  (5) | DK  (6) | N/A  (7) |
| --- | --- | --- | --- | --- | --- | --- | --- |
| 1. Do you share your thoughts and feelings with your mother? | ❑ | ❑ | ❑ | ❑ | ❑ | ❑ | ❑ |
| 2. Do you share your thoughts and feelings with your father? | ❑ | ❑ | ❑ | ❑ | ❑ | ❑ | ❑ |
| *3. If no parents,* do you share your thoughts and feelings with your guardian? | ❑ | ❑ | ❑ | ❑ | ❑ | ❑ | ❑ |
| 4. How often does your parent/guardian tell you or show you that they’re proud of you for something you have done? | ❑ | ❑ | ❑ | ❑ | ❑ | ❑ | ❑ |
| 5. Do your parents praise you when you work hard in school? | ❑ | ❑ | ❑ | ❑ | ❑ | ❑ | ❑ |
| 6. Now, thinking of the time when you were in school, how often did you enjoy being in school? | ❑ | ❑ | ❑ | ❑ | ❑ | ❑ | ❑ |
| 7. In your household, how often do people argue and fight? | ❑ | ❑ | ❑ | ❑ | ❑ | ❑ | ❑ |
| 8. Do you ever feel like family conflict is your fault? | ❑ | ❑ | ❑ | ❑ | ❑ | ❑ | ❑ |
| 9. How often do you attend religious services or activities? | ❑ | ❑ | ❑ | ❑ | ❑ | ❑ | ❑ |
| 10. During the past 30 days, how often have you smoked a cigarette, even one or two puffs? | ❑ | ❑ | ❑ | ❑ | ❑ | ❑ | ❑ |
| 11. During the past 30 days, how often have you had at least one drink of alcohol? | ❑ | ❑ | ❑ | ❑ | ❑ | ❑ | ❑ |
| 12. During the past 30 days, how often did you use *bhangi*? | ❑ | ❑ | ❑ | ❑ | ❑ | ❑ | ❑ |
| 13. During the past 30 days, how often were you in a physical fight? | ❑ | ❑ | ❑ | ❑ | ❑ | ❑ | ❑ |

|  | Yes  (1) | No  (2) | DK  (3) | N/A  (4) |
| --- | --- | --- | --- | --- |
| 14. Do you believe that it is important to be honest with your parents/guardians, even if they become upset or you get punished? | ❑ | ❑ | ❑ | ❑ |
| 15. Has anyone in your family ever had a severe alcohol or drug problem? | ❑ | ❑ | ❑ |  |
| 16. When you were in school, were your school grades better than the grades of most students in your class? | ❑ | ❑ | ❑ | ❑ |
| 17. Do you feel that there are lots of chances for students in your school to talk with a teacher one on one? | ❑ | ❑ | ❑ | ❑ |
| 18. Do you have a positive relationship with an adult in the community? | ❑ | ❑ | ❑ | ❑ |
| 19. When you were in school, if you had skipped school, would your parents/guardians know? | ❑ | ❑ | ❑ | ❑ |

|  | Excellent  (1) | Very Good  (2) | Average  (3) | Not Good  (4) | Poor  (5) | DK  (6) | N/A  (7) |
| --- | --- | --- | --- | --- | --- | --- | --- |
| 20. Putting them together, what were your grades like last year? | ❑ | ❑ | ❑ | ❑ | ❑ | ❑ | ❑ |

*Source: Communities That Care ® Youth Survey*

| **RISK/PROTECTIVE FACTORS MODULE (CONTD)** | Very Wrong  (1) | Somewhat Wrong  (2) | Not wrong at all  (3) | DK  (4) | N/A  (5) |
| --- | --- | --- | --- | --- | --- |
| 21. How wrong do your parents/guardians feel it would be for you to drink beer or other liquor? | ❑ | ❑ | ❑ | ❑ | ❑ |
| 22. How wrong do you think it is for someone your age to smoke *bhangi*? | ❑ | ❑ | ❑ | ❑ | ❑ |

|  | Age (Years) | Don’t Remember  (1) | DK  (2) | N/A  (3) |
| --- | --- | --- | --- | --- |
| 23. How old were you when you first began drinking alcoholic beverages regularly, that is, at least once or twice a month? |  |  | ❑ | ❑ |
| 24. How old were you when you first smoked *bhangi*? |  |  | ❑ | ❑ |

|  | No. | DK (999) |
| --- | --- | --- |
| 25. How many times have you done something dangerous because someone dared you to do it? |  | **❑** |
| 26. How many individuals have you had sexual intercourse with in your life? |  | **❑** |
| 27. Think of your four best friends. In the past year, how many of your best friends have dropped out of school? |  | **❑** |
| 28. Think of your four best friends. In the past year, how many of your best friends have smoked cigarettes? |  | **❑** |
| 29. Think again of your four best friends. In the past year, how many of your best friends do you think have had sex? |  | **❑** |

|  | Yes (1) | No (2) | DK (3) |
| --- | --- | --- | --- |
| 30. Have any of these friends smoked *bhangi* in the past year? | ❑ | ❑ | ❑ |
| 31. Have any of these friends stolen anything in the past year? | ❑ | ❑ | ❑ |
| 32. Have you stolen anything in the past year? | ❑ | ❑ | ❑ |
| 33. Have you ever had sexual intercourse? ***If Yes*,** how old were you when you first had sexual intercourse? ________ Yrs | ❑ | ❑ | ❑ |
| 34. If you ever had sexual intercourse, did you or your partner use a condom? | ❑ | ❑ | ❑ |
| 35. Are you sexually involved with anyone? ***If Yes,*** how old is this person? _________Yrs | ❑ | ❑ | ❑ |
| 36. Do you know what HIV/AIDS is? | ❑ | ❑ |  |
| 37. Do you believe that condoms reduce risk of HIV infection? | ❑ | ❑ | ❑ |
| 38. Do you often ignore rules that get in your way? | ❑ | ❑ | ❑ |
| 39. Do you often do things without thinking? | ❑ | ❑ | ❑ |

|  | Highly likely (1) | Likely (2) | Not Likely (3) | DK (4) |
| --- | --- | --- | --- | --- |
| 40. What are the chances you would be seen as cool if you engaged in sexual activities now? | ❑ | ❑ | ❑ | ❑ |
| 41. How likely do you think someone your age could get HIV/AIDS? | ❑ | ❑ | ❑ | ❑ |

1. **Please share any successes you have had in your life**
2. **In your opinion, what would you like to see from the families and community in regarding to caring and well-being of children?**
