# Supplementary material for: Rural-urban disparities in the nutritional status of younger adolescents in Tanzania
Source: PLoS One. 2021 Dec 20;16(12):e0261480. doi: 10.1371/journal.pone.0261480 (PMC8687541; doi:10.1371/journal.pone.0261480)
Supplement: S4 File — (DOC) [file pone.0261480.s004.doc]

**HOUSEHOLD SURVEY**

**ROUND 1**

**Household Baseline Survey**

**Questionnaire for Primary Care Giver – 1**

| 1. **Identification**   1.1 Household number **----------------------------------------------------------------------------** 1.2 Name of Household head -------------------------------------------------------------------- 1.3 Name of person interviewed-----------------------------------------------------------------  1.4 Name of Hamlet -----------------------------------------------------------------------------  1.5 Name of village/cluster ----------------------------------------------------------------------  1.6 Name of Ward ---------------------------------------------------------------------------------  1.7 Name of District ---------------------------------------------------------------------------  1.8 Name of Interviewer --------------------------------------------------------------------------  1.9 Name of Supervisor -------------------------------------------------------------------------- | **Comments** |
| --- | --- |

Date of Interview

| 1^st^ Time | | | 2^nd^ Time | | | 3^rd^ Time | | |
| --- | --- | --- | --- | --- | --- | --- | --- | --- |
| Day | Month | Year | Day | Month | Year | Day | Month | Year |
|  |  |  |  |  |  |  |  |  |

Start Time: __ __ __:__ __ __ Finish Time: __ __ __:__ __ __

**2a. Demographics and Income Sources: 2b.**What is the primary language spoken in the household? _______________

| Member ID | Can you please list the first names of all household members?**  Name | Sex  M…1  F….2 | How old is [name]?  Age* | | Relation to Household Head  (code) | Marital Status  (code) | Is s/he a valid HH member?**  Yes 1  No……. 2 | Religion  (code) | Ethnic Group  (code) | What is the last class completed in school? | Primary source of income for all individuals over 10 years  (code) |
| --- | --- | --- | --- | --- | --- | --- | --- | --- | --- | --- | --- |
|  |  |  | Year | Month |  |  |  |  |  |  |  |
| A | b | c | d | e | f | g | h | i | j | k | m |
| 1 |  |  |  |  |  |  |  |  |  |  |  |
| 2 |  |  |  |  |  |  |  |  |  |  |  |
| 3 |  |  |  |  |  |  |  |  |  |  |  |
| 4 |  |  |  |  |  |  |  |  |  |  |  |
| 5 |  |  |  |  |  |  |  |  |  |  |  |
| 6 |  |  |  |  |  |  |  |  |  |  |  |
| 7 |  |  |  |  |  |  |  |  |  |  |  |
| 8 |  |  |  |  |  |  |  |  |  |  |  |
| 9 |  |  |  |  |  |  |  |  |  |  |  |
| 10 |  |  |  |  |  |  |  |  |  |  |  |
| 11 |  |  |  |  |  |  |  |  |  |  |  |

* *The age of all children under tens should be calculated in years and months.*

**** A household member is a person who stays at least half of the week in the household and shares food from same pot.

| **Relation to Household Head code:**  Household head 1  Spouse 2  Son/daughter 3  Father/mother 4  Sister/brother 5  Son/daughter-in-law 6  Grand child 7  Brother/sister-in-law 8  Other relative 9  Non-relative 10 | **Marital Status code:**  Never married……...1  Married ….2  Separated ….3  Divorced ….4  Widow/widower ….5 | **Religion code:**  Christian………………1  Muslim………………..2  Traditional……………3  None………………….4  Other………………….5  **Ethnic Group code:**  WaKaguru………..1  WaSagara…………2  WaGogo…………..3  WaMaasai…………4  WaMang’ati……….5  Nyinginezo (taja)….6 | **Schooling/Educ Attain:**  No education………….1  Has not begun school…2  Kindergarten…………..3  Primary School………4  COBET……………….5  Secondary (1-4)………6  Secondary(5-6)……….7  Adult Education………8  Higher Education……..9  Haijulikani……………10 | **Primary Source of Income code:**  Farming, forestry, fishing………….……..1  Pastoralist…………………………………2  Non-agri day labor………….…………….3  Professional/Clerical……………………...4  Service (tailor, hairdresser, mechanic)……5  Domestic servant.…………………………6  Vendor (vegetable, products)……………..7  Beggar …………………………8  Mining…..………………………..………9  Commercial sex worker…………………10  Work in a bar…………………………….11  Not employed……………………………12  Other……………………………………..13 |
| --- | --- | --- | --- | --- |

**2c. Assets**

| Land holdings: *I would like to know something about land tenure in this community* | | | Code  Yes…1  No….2 | | Quantity  (acres) | How much is used for farming?  (acres) | | | Total Current Value (if sold this month)  (TSH) | |
| --- | --- | --- | --- | --- | --- | --- | --- | --- | --- | --- |
| a | | | b | | c | d | | | e | |
| i. Do you own any land? | | |  | |  |  | | |  | |
| ii. Do you rent out any farming land to other people? | | |  | |  |  | | |  | |
| iii. Do you rent farmland from someone else? | | |  | |  |  | | |  | |
| iv. Do you rent farmland from others for cash or in return for a portion of the crop (check applicable box)?    (1) (2) (3) (4) (5)  □ Cash only □ Portion of Crop □ Both cash and portion of crop □ Other (state) _______________ □ No Response | | | | | | | | | | |
|  | | | | **3.**  **Housing** | | | | | | |
| **Other Major Assets** Do you have the following assets? | Yes…1  No….2 | Quantity  (Number) |  | Rooms occupied by family (exclude cowshed, kitchen, etc)  (No. of rooms) | | | Roofing  Material  (code) | Outside Walls  Material  (code) | | Floor  Material  (code) |
| Fruit trees (miti) |  |  |  | a | | | b | c | | d |
| Bicycle (baiskeli) |  |  |  |  | | |  |  | |  |
| Boat (boti) |  |  |  |  | | | | | | |
| Car (gari)   | **Material Code:**  Brick/Concrete 1  Tin/corrugated iron 2  Thatch 3  Straw 4  Mud 5  Wood 6  Leaf 7  Other 8  Tile 9 | | --- | |  |  |  |  |  |  |  |  |  |  |
| Motorbike (pikipiki) |  |  |  |  |  |  |  |  |  |  |
| Chair(s) (kiti) |  |  |  |  |  |  |  |  |  |  |
| Table(s) (meza) |  |  |  |  |  |  |  |  |  |  |
| Clock (saa) |  |  |  |  |  |  |  |  |  |  |
| Bucket (ndoo) |  |  |  |  |  |  |  |  |  |  |
| Goats/Sheep/Donkey(s) (Mbuzi) |  |  |  |  |  |  |  |  |  |  |
| Cattle (N’gombe) |  |  |  |  |  |  |  |  |  |  |
| Poultry (chickens, duck .) (Kuku) |  |  |  |  |  |  |  |  |  |  |
| Pigs (nguruwe) |  |  |  |  |  |  |  |  |  |  |
| Mobile Telephone (simu) |  |  |  |  |  |  |  |  |  |  |
| Television (TV) |  |  |  |  |  |  |  |  |  |  |
| Radio/Tape player (radio) |  |  |  |  |  |  |  |  |  |  |

**4. Household Expenditure**

..

Now, I would like to ask you some questions about how much your household spends on health services and other things. *For all questions in this section report all values in local currency, whether paid in cash or in kind*

| Household Expenditure | TSH |
| --- | --- |
| a. In the past 30 days, how much did your household spend in total? |  |
| b. In the past 30 days, how much did your household spend on the following: | |
| b.1 Food, including such things as [maize, rice], meat, fruits, vegetables, and cooking oils. Include the value of any food that was produced and consumed by the household, and exclude alcohol, tobacco and restaurant meals. |  |
| b.2 Beer or other alcoholic beverages |  |
| b.3 Housing, gas, electricity, telephone, water, and heating fuel |  |
| b.4 Education fees and supplies |  |
| b.5 Health care costs, excluding any insurance reimbursements |  |
| b.6 Altogether, how much money do you owe others ( relatives, credit schemes, friends, etc)? |  |
| b.7 Other ____________________ |  |

*Source: World Health Survey, WHO*

The following questions should be directed to the primary food preparer

| **5. Food Frequency** | | Every Day  (1) | 3 - 6 times/wk  (2) | 1-2 times/wk  (3) | Less than once/wk  (4) | Never  (5) |
| --- | --- | --- | --- | --- | --- | --- |
| a. | In the last 7 days, how often did you eat less food than usual due to scarcity of food? | ❑ | ❑ | ❑ | ❑ | ❑ |
| b. | In the last 7 days, how often did you skip entire meals because there was no food? (not including days that you were sick) | ❑ | ❑ | ❑ | ❑ | ❑ |
| c. | In the last 7 days, how many times did you eat meat or fish? | ❑ | ❑ | ❑ | ❑ | ❑ |
| d. | In the last 7 days, how many times did you eat green leafy vegetables? | ❑ | ❑ | ❑ | ❑ | ❑ |
| e. | In the last 7 days, how many times did you eat pumpkin, carrots, mango, or papaya? | ❑ | ❑ | ❑ | ❑ | ❑ |

*Source: Tufts University FANTA Project*

**7a. Food Security (past 30 days)**

(1) (2) (3) (4) (5)

| ***In the last 30 days, during periods of when there was not enough food in the household or sufficient money to purchase food, how often did you do the following:*** | Very often  (Every day) | Often  (3-6x/wk) | Occasionally  (1-2x/wk) | Rarely  (<1 time/wk) | Never |
| --- | --- | --- | --- | --- | --- |
| a. Eat less preferred foods? | □ | □ | □ | □ | □ |
| 1. Borrow food or ask for help from friends or relatives? | □ | □ | □ | □ | □ |
| 1. Buy food on credit? | □ | □ | □ | □ | □ |
| 1. Harvest wild fruits or vegetables, hunt, or harvest immature crops early? | □ | □ | □ | □ | □ |
| 1. Eat seed stock held for the next season? | □ | □ | □ | □ | □ |
| 1. Send household members elsewhere to eat? | □ | □ | □ | □ | □ |
| g. Send household members to beg for food? | □ | □ | □ | □ | □ |
| h. Reduce portion size? | □ | □ | □ | □ | □ |
| i. Reduce adult consumption so that children can eat? | □ | □ | □ | □ | □ |
| j. Feed working members at the expense of non-working members? | □ | □ | □ | □ | □ |
| k. Reduce number of meals consumed per day? | □ | □ | □ | □ | □ |
| l. Skip meals for the entire day? | □ | □ | □ | □ | □ |
| m. Sell assets? | □ | □ | □ | □ | □ |
| n. Working as a day laborer for very little pay or a very small portion of food? | □ | □ | □ | □ | □ |
| o. Pawn assets? | □ | □ | □ | □ | □ |
| p. Steal? | □ | □ | □ | □ | □ |

*Source: Maxwell et al. Coping Strategies Index and Tufts University Hunger Module/FANTA Project*

**7b. Household Food Insecurity Access Scale (HFIAS)**

Now I have another set of similar questions to ask you. These are to help me make sure that I understand your food situation over the past 30 days.

| **1** **a.** In the past 30 days, did you worry that your household would not have enough food? | 1. Yes | 2. No **[GO TO 2a, skip 1b.]** | 97. Don’t know/not sure  99. Refused |
| --- | --- | --- | --- |
| **1 b.** How often did this happen? | 1. Often (more than ten times in the past 30 days)  2. Sometimes (three to ten times in the past 30 days)  3. Rarely (once or twice in the past 30 days) | | |
| **2** **a.** In the past 30 days, were you or any household member not able to eat the kinds of foods you preferred because of a lack of resources? | 1. Yes | 2. No **[GO TO 3a, skip 3b.]** | 97. Don’t know/not sure  99. Refused |
| **2 b.** How often did this happen? | 1. Often (more than ten times in the past 30 days)  2. Sometimes (three to ten times in the past 30 days)  3. Rarely (once or twice in the past 30 days) | | |
| **3 a.**  In the past 30 days, did you or any household member have to eat a limited variety of foods due to a lack of resources? | 1. Yes | 2. No **[GO TO 4a, skip 4b]** | 97. Don’t know/not sure  99. Refused |
| **3 b.** How often did this happen? | 1. Often (more than ten times in the past 30 days)  2. Sometimes (three to ten times in the past 30 days)  3. Rarely (once or twice in the past 30 days) | | |
| **4 a.** In the past 30 days, did you or any household member have to eat some foods that you really did not want to eat because of a lack of resources to obtain other types of food? | 1. Yes | 2. No **[GO TO 5a, skip 5b]** | 97. Don’t know/not sure  99. Refused |
| **4 b.** How often did this happen? | 1. Often (more than ten times in the past 30 days)  2. Sometimes (three to ten times in the past 30 days)  3. Rarely (once or twice in the past 30 days) | | |
| **5 a.** In the past 30 days, did you or any household member have to eat a smaller meal than you felt you needed because there was not enough food? | 1. Yes | 2. No **[GO TO 6a, skip 6b.]** | 97. Don’t know/not sure  99. Refused |
| **5 b.** How often did this happen? | 1. Often (more than ten times in the past 30 days)  2. Sometimes (three to ten times in the past 30 days)  3. Rarely (once or twice in the past 30 days) | | |
| **6 a.** In the past 30 days, did you or any household member have to eat fewer meals in a day because there was not enough food? | 1. Yes | 2. No **[GO TO 7a, skip 7b.]** | 97. Don’t know/not sure  99. Refused |
| **6 b.** How often did this happen? | 1. Often (more than ten times in the past 30 days)  2. Sometimes (three to ten times in the past 30 days)  3. Rarely (once or twice in the past 30 days) | | |
| **7 a.** In the past 30 days, was there ever no food to eat of any kind in your household because of lack of resources to get food? | 1. Yes | 2. No **[GO TO 8a, skip 8b]** | 97. Don’t know/not sure  99. Refused |
| **7 b.** How often did this happen? | 1. Often (more than ten times in the past 30 days)  2. Sometimes (three to ten times in the past 30 days)  3. Rarely (once or twice in the past 30 days) | | |
| **8 a.** In the past 30 days, did you or any household member go to sleep at night hungry because there was not enough food? | 1. Yes | 2. No **[GO TO 9a, skip 9b]** | 97. Don’t know/not sure  99. Refused |
| **8 b.** How often did this happen? | 1. Often (more than ten times in the past 30 days)  2. Sometimes (three to ten times in the past 30 days)  3. Rarely (once or twice in the past 30 days) | | |
| **9 a.** In the past 30 days, did you or any household member go a whole day and night without eating anything because there was not enough food? | 1. Yes | 2. No **[GO TO Section 10a, skip 10b]** | 97. Don’t know/not sure  99. Refused |
| **9 b.** How often did this happen? | 1. Often (more than ten times in the past 30 days)  2. Sometimes (three to ten times in the past 30 days)  3. Rarely (once or twice in the past 30 days) | | |

8a. Health Status

People in our community get sick from time to time. The following questions are about illness. Did any adult in the household have:

*Note: Complete Columns 1-10 for All Adult Household Members over 19 years, listed in “Demographics”.*

| Member ID | Persistent diarrhoea now or two or more episodes of acute diarrhoea in past 3 months  Definition: *3 + watery stools in a 24 hour period*  Yes…..1  No…...2  DK…..3 | Two or more episodes of cough, rapid breathing, wheezing, and fever in last 2 months?  Yes....1⇨ d  No…..2⇨ e  DK….3⇨e | Did person lose substantial weight during the past 12 months without regaining it back?  Yes...1⇨h  No…2⇨ i  DK...3⇨ i | Did person lose substantial weight during the past 12 months but regained most or all of it?  Yes....1  No…..2  DK….3 |
| --- | --- | --- | --- | --- |
| a | b | c | g | h |
|  |  |  |  |  |
|  |  |  |  |  |
|  |  |  |  |  |
|  |  |  |  |  |
|  |  |  |  |  |
|  |  |  |  |  |
|  |  |  |  |  |
|  |  |  |  |  |
|  |  |  |  |  |
|  |  |  |  |  |

Source: WHO

**8c. Health Status: Disability, HIV/AIDS, Malaria *Note: Complete Columns 1-11 for All Adult Household Members over 19 years, listed in “Demographics”.***

In the past year, have you or any adult in the household been sick/disabled for more than 1 month? Yes….1

No…..2 (if no, go to Column (e) in the box below)

| Member  ID | For how many days has this person been sick/disabled?  (No. of Days) | What type of sickness or disability does/did this person have? | Did this person have tuberculosis in the past year?  Yes…1  No….2  DK…3 | Did person have malaria in last 3 months?  Yes...1⇨g  No….2⇨h  DK…3⇨h | How did this person know s/he had malaria?  (code) | Did person receive medical treatment for his/her problem?  Yes…1⇨ j  No….2⇨i  DK…3⇨i  N/A…4⇨i | If no, was this due to lack of money?  Yes…1  No….2  DK…3  N/A..4 | Was this person an income earner before becoming sick/disabled?  Yes….1  No…..2  No Resp…3  N/A…..….4 | Is person earning less income after becoming sick/disabled?  Yes….1  No…..2  No Resp….3  N/A………4 |
| --- | --- | --- | --- | --- | --- | --- | --- | --- | --- |
| a | b | c | e | f | g | h | i | j | k |
|  |  |  |  |  |  |  |  |  |  |
|  |  |  |  |  |  |  |  |  |  |
|  |  |  |  |  |  |  |  |  |  |
|  |  |  |  |  |  |  |  |  |  |
|  |  |  |  |  |  |  |  |  |  |
|  |  |  |  |  |  |  |  |  |  |
|  |  |  |  |  |  |  |  |  |  |
|  |  |  |  |  |  |  |  |  |  |
|  |  |  |  |  |  |  |  |  |  |

#### Source: Tufts University/FANTA Project

| **How did person know it was HIV/AIDS code :**  Doctor/clinic/VCT diagnosed with blood test...……..1  Doctor/clinic/VCT diagnosed w/o blood test………..2  Other provider (traditional healer, etc) diagnosed……3  Someone who is not a provider told me….….………4  I knew because of the symptoms…………………….5  Other…………………………………………………6 | **How did person know it was Malaria code :** Doctor/clinic diagnosed with blood test...……………1  Doctor/clinic diagnosed w/o blood test………………2  Other provider (traditional healer, etc) diagnosed……3  Someone who is not a provider told me….….……….4  I knew because of the symptoms…………………….5  Other………………………………………………….6 |
| --- | --- |
